# Analysis of striatal transcriptome in mice overexpressing human wild-type alpha-synuclein supports synaptic dysfunction and suggests mechanisms of neuroprotection for striatal neurons

**DOI:** 10.1186/1750-1326-6-83

**Published:** 2011-12-13

**Authors:** Yofre Cabeza-Arvelaiz, Sheila M Fleming, Franziska Richter, Eliezer Masliah, Marie-Francoise Chesselet, Robert H Schiestl

**Affiliations:** 1 Department of Pathology and Environmental Health Sciences, The Geffen School of Medicine and School of Public Health, University of California, Los Angeles, 650 Charles E. Young Dr. S, CHS 71-295; Los Angeles, CA 90095, USA; 2 Department of Neurology, The Geffen School of Medicine, University of California, Los Angeles, 710 Westwood plaza, Los Angeles, CA 90095, USA; 3 Department of Neurosciences, University of California, San Diego; 9500 Gilman Drive, La Jolla, CA 92093, USA

**Keywords:** α-synuclein, apoptosis, neuroprotection, Parkinson's disease, Alzheimer's disease, synaptic plasticity, vesicle release, diabetes

## Abstract

**Background:**

Alpha synuclein (SNCA) has been linked to neurodegenerative diseases (synucleinopathies) that include Parkinson's disease (PD). Although the primary neurodegeneration in PD involves nigrostriatal dopaminergic neurons, more extensive yet regionally selective neurodegeneration is observed in other synucleinopathies. Furthermore, *SNCA *is ubiquitously expressed in neurons and numerous neuronal systems are dysfunctional in PD. Therefore it is of interest to understand how overexpression of SNCA affects neuronal function in regions not directly targeted for neurodegeneration in PD.

**Results:**

The present study investigated the consequences of SNCA overexpression on cellular processes and functions in the striatum of mice overexpressing wild-type, human *SNCA *under the Thy1 promoter (Thy1-aSyn mice) by transcriptome analysis. The analysis revealed alterations in multiple biological processes in the striatum of Thy1-aSyn mice, including synaptic plasticity, signaling, transcription, apoptosis, and neurogenesis.

**Conclusion:**

The results support a key role for SNCA in synaptic function and revealed an apoptotic signature in Thy1-aSyn mice, which together with specific alterations of neuroprotective genes suggest the activation of adaptive compensatory mechanisms that may protect striatal neurons in conditions of neuronal overexpression of SNCA.

## Background

Abnormal accumulation of the pre-synaptic protein α-synuclein (SNCA) is a hallmark of several neurodegenerative disorders including the second most frequent neurodegenerative disease Parkinson's disease (PD) [1]. Neurodegeneration in PD is predominant in the substantia nigra pars compacta (SNc), but cell loss and Lewy Body (LB) formation also occur in other brain and peripheral tissues [2]. Familial forms of PD have been linked to mutations in *SNCA*, and also to multiplications of the locus encompassing the *SNCA *gene, which lead to increased levels of SNCA expression indicating that the wild-type (wt) protein can be pathogenic if produced in excess [3]. Furthermore, Genome Wide Association Studies (GWAS) have consistently identified the *SNCA *gene as most associated with PD risk [4]. In most synucleinopathies, SNCA aggregates form in neurons [5]. Transgenic (tg) murine models expressing human *SNCA *under neuronal promoters reproduce some phenotypic features of PD such as inclusion formation, motor and non-motor impairments, loss of striatal dopamine (DA), and, in a few tg lines, nigrostriatal degeneration (For review see: [6,7]). Mice expressing human wild-type *SNCA *under the Thy1 promoter (Thy1-aSyn mice) express high level of mRNA and protein in neurons throughout the brain [8] and develop proteinase K-resistant SNCA aggregates [9,10]. These mice show a 40% loss of DA in the striatum by 14 months of age [11]. We have shown that these mice display early and progressive sensorimotor anomalies, abnormal response to stimulants, olfactory deficits and digestive dysfunction before the loss of striatal DA [6,7,10,12-14]. In addition, they show profound anomalies of cortico-striatal transmission [15,16], suggesting alterations within motor cortico-subcortical loops. Whole transcriptome analysis provides a valuable alternative approach for the detection of key changes that might not be practical to attempt by directed single-gene/protein approaches. Previous studies have evaluated alterations in gene expression patterns in cells from SNc and striatal tissues from transgenic mice overexpressing SNCA in the SNc under different promoters [17,18]. However, little is known of the effects of SNCA overexpression in the striatum itself, the region that contains the axon terminals of dopaminergic (DArgic) neurons and mediates the behavioral effects of DA depletion in PD.

To gain a better understanding of the consequences of excessive SNCA expression on basal ganglia function, we performed transcriptome analysis of striatal tissue from male Thy1-aSyn-mice and wt littermates. The data further support a key role for SNCA in synaptic function and also reveal alterations in multiple biological processes including signaling, apoptosis, and neurogenesis, which may be related to both functional deficits resulting from SNCA overexpression and the sparing of striatal neurons in most synucleinopathies.

## Results and discussion

### Differentially regulated genes and functional categories

To understand how increased SNCA expression causes neuronal cellular dysfunction, we analyzed gene expression in the striatum, the major target of the nigrostriatal DArgic projections and a brain region that plays a key role in the control of motor, affective and cognitive functions but does not degenerate in PD [19]. We elected to examine changes in gene expression in the striatum of 6-month-old mice because, as shown in our previous publications [9-12,14], the Thy1-aSyn mice begin the show progressive behavioral impairments from 2 months of age but do not show significant DArgic loss in the striatum until 14 months of age [11]. Therefore, the time point chosen for these studies corresponds to a pre-manifest phase of PD, when neuronal dysfunction due to SNCA pathology is present, but DA is not yet lost.

In order to have sufficient mRNA to generate transcript concentrations in the range required to be detected safely above background levels, total RNA from groups of 6 tg Thy1-aSyn or 6 wt mice were pooled. Pooling has the added advantage that it minimizes individual variations as a source of gene-expression variance, which impacts the identification of differentially expressed genes by DNA microarrays [20]. Genes differentially regulated in Thy1-aSyn mice compared to wt littermates were identified by whole transcriptome analysis. The relative fold increased expression of human *SNCA *in the Thy1-aSyn mice used, as assessed by qRT-PCR, is shown in Table 1. The *SNCA *primer set used hybridized to human *SNCA *and mouse *Snca *mRNAs, albeit less efficiently to the latter, therefore showing relative differences in total *SNCA *mRNA expression between wt and tg mice. However, the very high level of expression of *SNCA *mRNA only results in a 50% increased level of total SNCA protein in this region in the Thy1-aSyn mice based on quantitative immunohistochemical analysis (Franich, Richter, and Chesselet unpublished observations). This suggests that the model is suitable to evaluate the effects of excess SNCA in the range observed in humans with *SNCA *gene duplication leading to familial forms of PD [3]. Notably, our mouse microarray data rule out a loss of transcriptional expression of endogenous *Snca *in Thy1-aSyn mice (Table 1). The quality of the data from the microarray was assessed (see Table 1 and Additional file 1 Figure S1) by inspecting: (1) the percentage of genes called present (include all changed genes p < 0.005), which was comparable and above 50% for both mice groups (wt and tg), and (2) the mean signal intensity, which was quite similar for both mice groups. After pairwise comparison, a list of 833 genes altered in Thy1-aSyn mice was generated. Further filtering to remove genes with fold change below 1.5, or that were called absent in both groups reduced the list to 263 genes, which was reduced further to the final 233 differentially expressed genes after removal of redundant genes. The number of upregulated genes was 96 genes whereas 137 genes were downregulated (Table 1), indicating that SNCA overexpression effects are mediated more prominently through negative regulation of transcription. The complete list of differentially expressed genes with respective test data is available (see Additional file 2 Table S1). To identify functional categories overrepresented in the lists of genes altered in Thy1-aSyn mice we used DAVID [21], which implements the Gene Ontology (GO) terms in three structured ontologies that relate gene products on the basis of their associated biological processes (BPs), cellular components (CC), and molecular functions (MFs); it also incorporates the Genetic Association Database (GAD) of human diseases, which allows for the identification of genes associated with human diseases [22]. The significant overrepresentation in GO and GAD of the 224 genes that had meaningful annotations pointed to alterations in gene expression between Thy1-aSyn and wt mice. These 224 genes were categorized into BPs, CCs and MFs and multiple overlapping GO terms (referred to as functional categories hereafter) and diseases were identified; 28 representative and significantly represented groups (p < 0.05) were selected and are listed in Table 2; these groups were then organized into six *ad hoc *function-related groups, with considerable overlap between them, and are shown in Table 2. Each of these groups will be discussed separately below.

**Table 1:** Array data quality control and differentially-regulated genes in ASO mice

Mice Genotype	*SNCA *expression		Data quality control			Altered genes		
	
	species	fold change (mean ± sd)	source	**% calls (present**)	intensity (mean ± sd)	up	down	total
**ASO**	**m*Snca^a^***	0.9 ± 0.1	**All ^c^**	57.5	197 ± 445	349	484	833
								
	**h*SNCA^b^***	155 ± 85	**D.E.G.^d^**	84.1	220 ± 271	96	137	233

**wt**	**h*SNCA***	1.1 ± 0.5	**All ^c^**	58.6	194 ± 425	0	0	0
								
	**m*Snca***	1.0 ± 0.0	**D.E.G.^d^**	91.3	223 ± 295	0	0	0

**Abbreviations: **ASO: α-Synuclein (*SNCA*) overexpressing; wt; wild type; D.E.G.: differentially expressed gene

**Notes: a**- m*Snca *detected by 3 probe sets in theMoe-430A chip; **b**- h*SNCA *measured by qRT-PCR in 3 wt and 3 ASO mice (Figure 2B); **c**- Include all changed genes P < 0.005; **d**- Selection criteria for D.E.G. (1) gene is present in at least one of the mice groups examined and (2) Fold change > 1.52 (Signal log2 ratio > 0.6), p < 0.005.

**Table 2:** Enriched functional categories for genes differentially regulated by α-synuclein

Database identifier	Functional category (Enriched GO and GAD terms)	Number of changed genes		
		
		up	down	pvalue
**MF **SP_PIR_KEY	phosphoprotein	49	64	8E-07
**BP **GO:0007242	intracellular signaling cascade	11	8	5E-02
**BP **KEGG_PATH	mmu04010:MAPK signaling pathway	5	4	3E-02
**BP **KEGG_PATH	mmu04020:Calcium signaling pathway	3	5	1E-02

**CC **GO:0045202	synapse	7	10	3E-06
**CC **GO:0005856	cytoskeleton	12	10	4E-02
**CC **GO:0031410	cytoplasmic vesicle/vesicle-mediated transport^a^	8	7	1E-02
**BP **GO:0048167	regulation of synaptic plasticity	3	3	4E-04
**BP **GO:0007267	cell-cell signaling	4	5	3E-02
**BP **GO:0006897	endocytosis	4	3	5E-02

**BP **GO:0045941	positive regulation of transcription	5	10	3E-03
**BP **GO:0042127	regulation of cell proliferation	8	9	2E-03
**MF **GO:0005520	IGF binding/growth factor binding^b^	1	4	7E-03
**BP **GO:0051259	protein oligomerization	2	3	1E-02
**BP **GO:0042981	regulation of apoptosis	6	11	2E-03
**BP **GO:0007610	behavior	10	13	7E-04

**BP **GO:0007626	locomotory behavior	5	8	4E-03
**BP **GO:0008344	adult locomotory behavior	1	4	8E-03
**BP **GO:0007631	feeding behavior	5	7	5E-04
**BP **GO:0001975	response to amphetamine	2	1	2E-02
**BP **GO:0030146	diuresis/excretion^c^	3	1	2E-03
**MF **GO:0005179	hormone activity	1	5	2E-02

**BP **GO:0016125	sterol metabolic process/cholesterol metabolism^d^	5	4	2E-02
**BP **GO:0030182	neuron differentiation	10	10	9E-07
**BP **GO:0031175	neuron projection development	7	6	2E-05
**BP **GO:0001944	vasculature development	2	6	4E-02

**D **G_A_DB_D	neurological diseases	4	5	5E-02
**D **G_A_DB_D	diabetes, types 1 and 2	3	6	5E-02

**Abbreviations: **D: disease; G_A_DB_D:Genetic_Association_DataBase_D; GO: Gene Ontology; BP: Biological process; MF: Molecular function; CC: Cell. component; KEGG_PATH: KEGG_PATHWAY; SP_PIR_KEY: SP_PIR_KEYWORDS;

**Notes**: **a- **GO:0016192; **b- **GO:0019838; **c- **GO:0007588; d- SP_PIR_KEYWORDS: cholesterol metabolism.

### Validation of Microarray results by alternative methods

**Figure 1:**
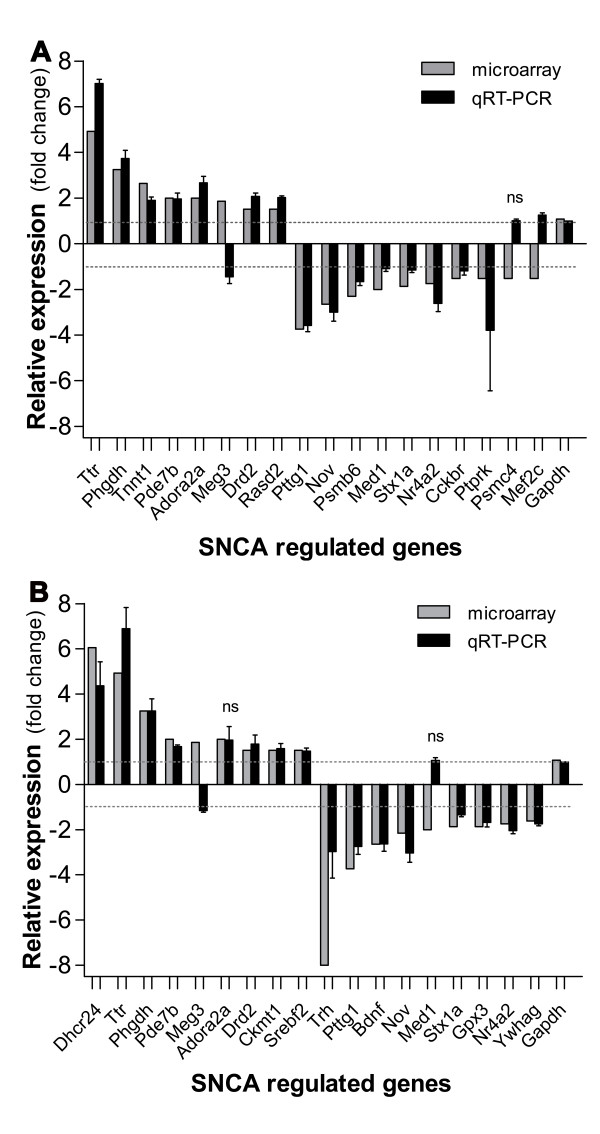
**Corroboration of microarray results for differentially expressed genes**. Confirmation of the changes of selected microarray differentially expressed genes by comparison to the changes assessed by quantitative real-time polymerase chain reaction (qRT-PCR) using: A- pooled RNA from wild-type (wt) and α-synuclein (*SNCA*) overexpressing transgenic (tg) mice groups; B- RNA from individual wt and tg mice. The qRT-PCR average fold changes of selected genes in tg mice striata normalized to *Gapdh *(Glyceraldehyde-3-phosphate dehydrogenase), and relative to expression in wt mice (dotted line) are shown (mean ± SEM); ns (not significantly different expression between tg and wt mice, using a t-test, n = 3, p > 0.05); SEM values in (A) show qRT-PCR technical variation in, and in (B) show variation between mice plus qRT-PCR technical variation). Pearson's test found significant correlation between microarray and qRT-PCR results in A (r^2 ^= 0.7288, p < 0.0001) and B (r^2 ^= 0.7354, p < 0.0001). *Ttr *(transthyretin), *Phgdh *(3-phosphoglycerate dehydrogenase), *Pde7b *(phosphodiesterases 7b), *Tnn1 *(troponin t1), *Adora2a *(adenosine A2a receptor), *Meg3 *(Maternally expressed 3), *Drd2 *(dopamine receptor 2), *Rasd2 *(rasD family, member 2), *Psmb6 *(proteasome (macropain) subunit, β 6), *Med1 *(mediator complex subunit), *Ptprk *(protein tyrosine phosphatase, receptor, K), *Pttg1 *(pituitary tumor-transforming 1), *Nov *(Nephroblastoma Overexpressed), *Pparbp *(peroxisome proliferator activated receptor binding protein), *Stx1a *(syntaxin 1a), *Nr4a2, Psmc4 *(proteasome (macropain) 26S subunit, ATPase, 4), *Mef2c *(myocyte enhancer factor 2c), *Cckbr *(cholecystokinin B receptor), *Dhcr24 *(24-dehydrocholesterol reductase), *Ckmt1 *(creatine kinase, mitochondrial 1), *Srebf2 *(sterol regulatory element-binding factor 2), *Trh *(thyrotropin-releasing hormone), *Bdnf *(brain derived growth factor), *Gpx3 *(glutathione peroxidase 3), *Ywhag *(Y-3-monooxygenase/W 5-monooxygenase activation protein, γ polypeptide).

**Figure 2:**
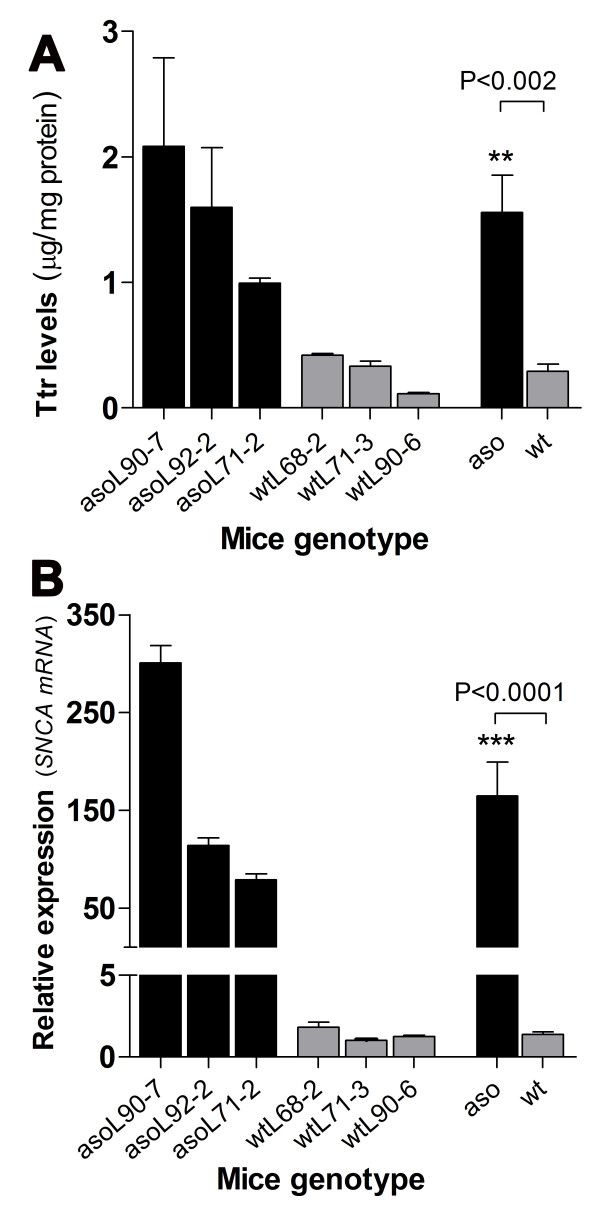
**Validation of microarray and qRT-PCR results for the highly expressed transthyretin (*Ttr*) gene**. The confirmation of the differential expression of *Ttr *was achieved by measuring striatum Ttr protein levels by ELISA in individual wt (wild-type) mice and aso (α-synuclein (*SNCA*) overexpressing or Thy1-aSyn mice). A- The average Ttr protein levels in the striata from Thy1-aSyn mice and wt mice groups are shown (mean + SEM, **p < 0.01, non-parametric Mann-Whitney t-test, aso vs wt, n = 3 per group); levels of Ttr in each individual mouse are also shown to illustrate variation between mice (error bars represent SEM showing technical variations in triplicate ELISA measurements). B- The average human *SNCA *mRNA levels in the striata from Thy1-aSyn mice and wt mice groups determined by qRT-PCR, normalized to *Gapdh*, and relative to the levels of endogenous *Snca *mRNA in the wt mice that partially hybridize to the human *SNCA *primers are shown (mean + SEM, ***p < 0.001, non-parametric Mann-Whitney t-test, aso vs wt, n = 3 per group); levels of *SNCA *mRNA in each individual mouse are also shown to illustrate variation between mice (error bars represent SEM showing technical variation in triplicate qRT-PCR analyses. Pearson's test found significant correlation (r^2 ^= 0.6791, p < 0.043) between the two measured expression values in A and B.

**Figure 3:**
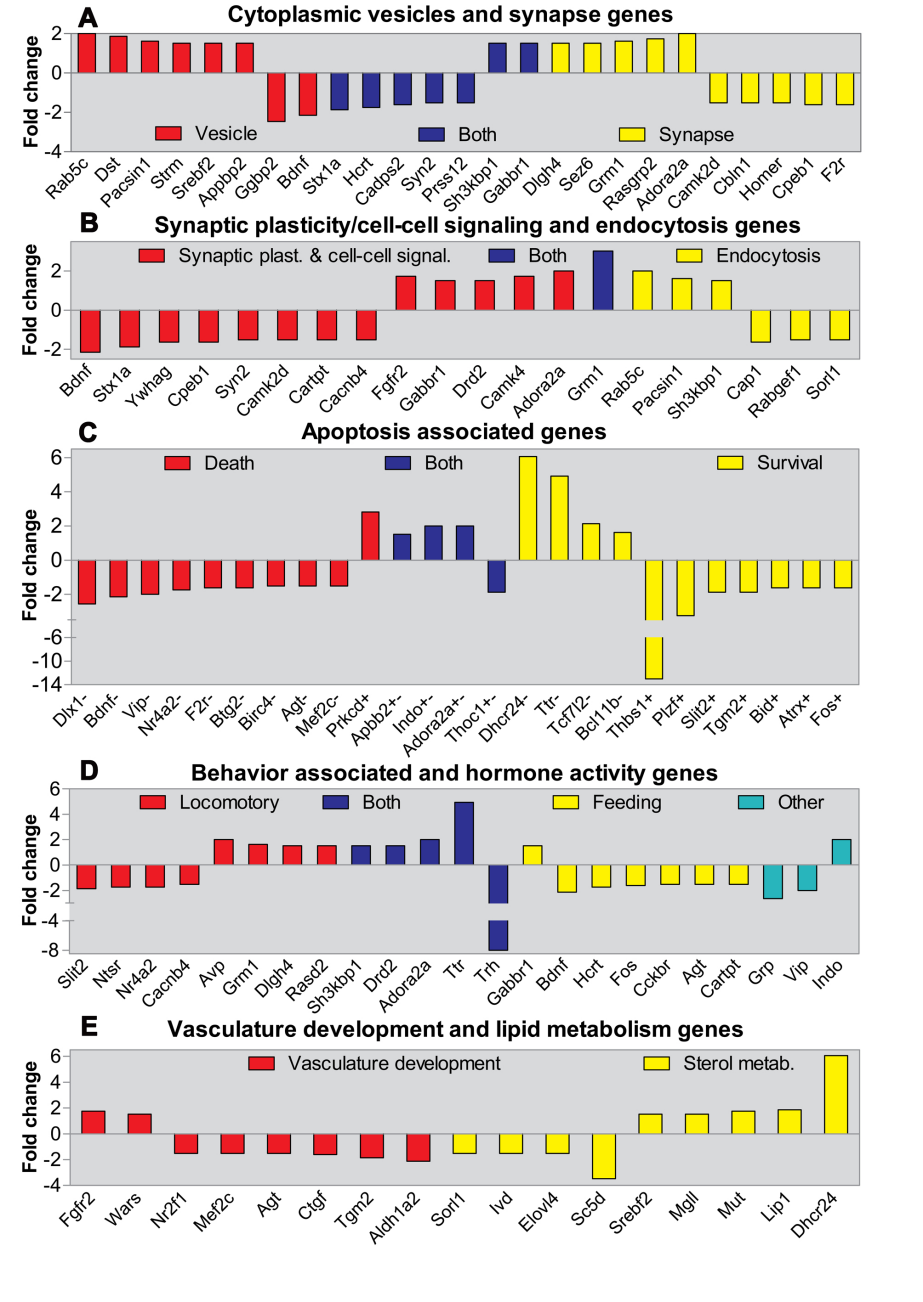
**Gene expression patterns changes in cellular processes affected by overexpression of *SNCA***. Functional categories overrepresented in the lists of genes differentially expressed in Thy1-aSyn mice were identified using the Gene ontology (GO) database, which implements the GO terms in three structured ontologies relating gene products on the basis of their associated biological processes, cellular components and molecular functions; multiple overlapping GO terms (referred to as functional categories) were identified (Table 2) (p < 0.05). Shown here are the genes expression pattern (fold changes in Thy1-aSyn mice relative to wt mice) in representative functional categories deemed highly relevant to PD pathogenesis. A) cytoplasmic vesicle/vesicle-mediated transport (GO:0031410, GO:0016192) and synapse (GO:0045202); B) regulation of synaptic plasticity/cell-cell signaling (GO:0048167, GO:0007267) and endocytosis (GO:0006897); C) regulation of apoptosis (GO:0042981) plus additional apoptosis regulators identified through data-mining; the sign + after the gene symbol indicates the gene is proapoptotic, the sign - indicates the gene antiapoptotic term and the signs +- or the term "Both" refers to ambivalent genes that can positively and negatively regulate apoptosis; D) behavior (GO:0007610) and hormone activity (GO:0005179); E) vasculature development (GO:0001944) and sterol metabolic process/cholesterol metabolism (GO:0016125, SP PIR_KEYWORDS: cholesterol metabolism). Most genes name descriptions can be found in the text; otherwise in Additional file 2 Table S1.

Microarray data was validated by qRT-PCR analysis of a total of 25 selected genes. 18 selected genes were validated using pooled total RNAs from Thy1-aSyn and wt mice (Figure 1A). Similarly, 11 of these 18 genes plus 7 other different genes were also validated using total RNA from a separate cohort of individual mice (n = 3) to assess biological variability in gene expression (Figure 1B). In addition, the microarray and qRT-PCR results for the highly expressed transthyretin (*Ttr*) gene were validated using ELISA [23] to measure Ttr protein levels in the same cohort of individual mice (Figure 2A) and compared to the levels of *SNCA *gene expression (Figure 2B). Genes were selected for PCR validation mainly for the following reasons: they encompass low, moderate and high intensity signal genes and they form part of one or more of the functional categories (listed in Table 2) that were significantly over-represented in the list of differentially-expressed genes. Although not exhaustive, we consider this list of genes representative of the total gene expression profile in affected pathways. In particular, it encompasses at least 3 genes from those overlapping pathways that have been associated with neurodegeneration and the PD phenotype, summarized in Figure 3. The qRT-PCR-analyzed genes are distributed among the functional categories as follows: A- Vesicles and Synapse (*Srebf2, Bdnf, Stx1A, Adora2A*), B- Synaptic function (*Bdnf, Stx1A, Ywhag, Drd2, Adora2A, Pde7b*), C- Apoptosis (*Bdnf, Nr4a2, Mef2c, Adora2A, Dhcr24, Ttr*), D- Behavior (*Nr4a2, Rasd2, Drd2, Adora2A, Ttr, Trh, Bdnf, Cckbr*), and E- Vasculature and lipid metabolism (*Mef2c, Srebf2, Dhcr24*); and a few other categories, which for reasons of space we chose not to show in Figure 3: Neurogenesis (*Bdnf, Nr4a2, Phgdh*), Cell cycle (*Pttg1, Ptprk, Dhcr24, Ywhag*), Signaling (*Ptprk, Pde7b, Drd2, Adora2A, Trh, Ywhag*), Cell growth/proliferation (*Bdnf, Meg3, Nov, Ptprk*), Transcription (*Med1, Mef2c, Nr4a2, Srebf2*), Response to oxidative stress (*Gpx3, Dhcr24, Nr4a2*). In addition, we checked 4 "orphan" genes, not associated with any significant category, but which could still be relevant to PD pathogenesis including, *Ckmt1 *(Creatine metabolism and energy generation), *Tnnt1 *(Muscle contraction), *Psb6 *and *Psmc4 *(Proteasome and cell cycle check point). Validation in individual mice was done on blocks of striatal tissue carefully dissected out from tissue slices to eliminate potential contamination from adjacent tissue, including the subventricular zone and choroid plexus. The expression of 3 of these genes in Thy1-aSyn; namely, *Psmc4 *(Proteasome (prosome, macropain) 26S subunit, ATPase, 4) in pooled samples (Figure 1A) plus *Adora2a *(Adenosine A2a receptor) and *Med1 *(mediator complex subunit) in non-pooled samples (Figure 1B) did not differ significantly from wt mice by qRT-PCR and therefore disagree with the microarray results. In addition, *Meg3 *(Maternally expressed 3, imprinted maternally expressed untranslated mRNA) and *Mef2c *(myocyte enhancer factor 2c) changed in opposite directions by the two methods. Nevertheless, the expression of most genes in the Thy1-aSyn mice group with both pooled and non-pooled RNA was generally similar between the microarray and qRT-PCR analyses, as indicated by the assessment of correlation using the Pearson's test which found strong and significant correlation between microarray and qRT-PCR measured expression values in A (r^2 ^= 0.7288, p < 0.0001) and B (r^2 ^= 0.7354, p < 0.0001). These data suggest that the direction and magnitude of change of gene expression levels (i.e. either up or down regulation) is accurately predicted by comparison of microarray expression values.

Because the main purpose of this study is to assess changes in transcriptome, individual changes were not systematically validated at the protein level. However, we did measure the protein levels of transthyretin because upregulation of this gene has been associated with neuroprotection in Alzheimer disease [24], and its high induction in the striatum of Thy1-aSyn mice was unexpected and of potential functional significance. The results, shown in Figure 2A, indicate that Ttr protein levels are significantly elevated in individual Thy1-aSyn mice and appear to be associated with the levels of *SNCA *mRNA as indicated by the assessment of correlation using the Pearson's test which found significant correlation (r^2 ^= 0.6791, p < 0.043) between the levels of Ttr protein (Figure 2A) and *SNCA *mRNA levels (Figure 2B).

### SNCA overexpression affects signaling pathways associated with the pathophysiology of neurodegenerative diseases

The first group of functional categories influenced by *SNCA *overexpression in Table 2 encompasses major signal transduction pathways such as the mitogen-activated protein kinases (MAPK) and Ca2+ signaling cascades that regulate multiple cellular processes [25-27]. Protein phosphorylation is a preponderant regulatory mechanism of signal transduction cascades in eukaryotic cells that is catalyzed by kinases and reversed by protein phosphatases [26]. Not surprisingly, half of the genes affected in Thy1-aSyn mice are phosphoproteins including kinases, phosphatases and phosphodiesterases (PDEs). Two PDEs that were elevated by 2-fold (Table S1) in Thy1-aSyn mice, Pde7b and Pde10a, are predominantly expressed in the striatum and have been associated with DArgic signaling [28,29], indicating that SNCA may influence post-synaptic DArgic signaling in striatal neurons through these enzymes. Interestingly, functional alterations in post-synaptic DArgic signaling have been detected in the striatum of these mice [11,13]. Additionally, members of the main signal transduction systems that mediate long-term potentiation and memory were affected in Thy1-aSyn mice. Notably, the synaptic Ca2+ signaling system seems altered, with reduced expression of *Camk2d *(Ca2+/calmodulin-dependent protein kinase II, delta) [30] (Figure 3A), *Cacnb4 *(Ca2+ channel, voltage-dependent, beta 4 subunit) (Figure 3B), and the (Ca2+)-activated transcription factor *Mef2c *that is known to promote neuronal survival [31] (Figure 3C), and increased expression of *Camk4 *(Ca2+/calmodulin-dependent protein kinase IV) (Figure 3B) [32,33]. Although inspection of the MAPK pathway genes affected in Thy1-aSyn mice does not allow us to surmise the status of this pathway, the decreased expression of the Fos gene (Figure 3C, D) in these mice is consistent with previously reported suppression of the MAPK pathway by elevated SNCA [34]. Thus, Ca2+ homeostasis and DArgic signaling may be affected in the striatum of Thy1-aSyn mice.

Both behavioral and electrophysiological responses to amphetamine, an indirect DA agonist, are profoundly altered in Thy1-aSyn mice [11,13]. Interference with the amphetamine response in Thy1-aSyn mice may be mediated by the decrease of *Cartpt *and the increase of *Rasd2 *(rasD family, member 2, a.k.a. Rhes) (Figure 3D). *Cartpt *is upregulated in the striatum by amphetamine [35], and *Rasd2 *has been shown to inhibit the stereotypy induced by co-activation of Drd1/Drd2 and by the Drd2 receptor alone [36], reminiscent of the decreased amphetamine-induced stereotypies observed in Thy1-aSyn mice [13].

### Alterations in the expression of synaptic vesicle cycle and synaptic plasticity associated genes

The second group in Table 2 includes cellular mechanisms comprising genes encoding for components of the synapse, cytoplasmic vesicles and cytoskeleton, which participate in biological processes such as the synaptic vesicle cycle, endocytosis, and synaptic plasticity, whose deregulation is highly relevant to the pathophysiology of neurodegenerative diseases [37,38]. At synapses, the synaptic vesicle release cycle is a tightly regulated cascade of events that involves the interplay of several proteins, including cytoskeleton components, to control synaptic vesicle mobilization between functional pools prior to their release [38].

The results from our study suggest that SNCA may control these processes through the transcriptional regulation of genes (Figure 3A, B) whose encoded proteins influence vesicle release and recycling such as the downregulation of *Syn2 *(Synapsin II), and *Cadps2 *((Ca2+)-dependent activator protein for secretion 2), which positively regulate these processes [39,40]. Indeed, study of mice lacking *Snca *showed that *Syn2 *is required to maintain normal numbers of synaptic vesicles and to regulate synaptic plasticity (for review see: [39]); Cadps2 has been implicated as a calcium sensor involved in constitutive vesicle trafficking and secretion [40]. Hence, *Syn2 *and *Cadps2 *downregulation in Thy1-aSyn mice is consistent with recent compelling evidence showing that modest SNCA overexpression markedly inhibits neurotransmitter release by a reduction in size of the synaptic vesicle recycling pool and through a defect in the reclustering of synaptic vesicles after endocytosis [41], and suggests that this specific SNCA effect may involve transcriptional repression of these genes. Support for a role of SNCA in regulating endogenous presynaptic proteins comes from a recent study in cultured neurons from *tg *mice overexpressing modest level of SNCA [42], which suggests that the decrease of endogenous presynaptic proteins by excessive SNCA may lead to functional impairments at synapses causing vesicle release inhibition [41]. Interestingly, synapsin was the most diminished of the four proteins analyzed in the study by Scott et al. [42], which is consistent with our results (*Syn2 *was downregulated).

Endocytosis genes that were altered in Thy1-aSyn mice encode for proteins involved in both the clathrin-mediated endocytosis (CME), as well as in the activity-dependent bulk endocytosis (ADBE), endosomal recycling and early endosome. These include, *Pacsin1 *(Protein kinase C and casein kinase substrate in neurons 1), and *Sorl1 *(Sortilin-related receptor, LDLR class A), (Figure 3B). Pacsin1 has recently been shown to be essential for the ADBE that is triggered during increased neuronal activity [43] and both ADBE and clathrin-mediated endocytosis contribute to the replenishment of synaptic vesicles [44]. Thus Pacsin1 upregulation in Thy1-aSyn mice may lead to increased neurotransmitter synaptic vesicles, which could provide a compensatory mechanism for the detrimental effects of excess SNCA on synaptic vesicles observed by Nemani et al. [41]. SORL1 has been shown to guide trafficking of amyloid precursor protein (APP) into recycling (endocytic) pathways and its decreased expression leads to the sorting of APP into amyloid generating compartments. This suggests that changes in SORL1 expression or function may be mechanistically involved in Alzheimer's disease (AD) pathogenesis [45]. The downregulation of *Sorl1 *in the Thy1-aSyn mice is of particular interest in view of compelling evidence indicating that amyloid and SNCA interact in vivo and promote each other aggregation and accumulation [46].

Genes involved in post-synaptic neurotransmitter signaling in striatal neurons were also significantly affected in Thy1-aSyn mice (Figure 3B). The expression of the receptor genes *Drd2 *(DA receptor D2), *Grm1 *(Glutamate receptor, metabotropic 1), *Adora2a *and *Gabbr1 *(γ-aminobutyric acid (GABA-B) receptor, 1) was upregulated in Thy1-aSyn mice. Adora2a is particularly interesting because caffeine, an adenosine A2a receptor antagonist, is protective against PD and Adora2a antagonists are developed for treating PD [47]. In addition, *Sh3kbp1 *(SH3-domain kinase binding protein 1, a.k.a. Cin85), also increased in Thy1-aSyn mice (Figure 3B), has been shown to positively regulate Drd2 endocytosis in the striatum [48]. Hence, its increased expression may increase Drd2 endocytosis in striatal neurons in response to DA stimulation and could explain abnormal responses to DA receptor stimulation in striatal slices of the Thy1-aSyn mice [11].

### SNCA overexpression triggers molecular changes that may underlie neuroprotection

The third group in Table 2 includes biological processes involved in the regulation of fundamental cellular mechanisms for cell transcription, cell proliferation, protein degradation and apoptosis. The expression balance of transcription genes in the Thy1-aSyn mice is tilted towards repression, as indicated by the repression of more genes that positively regulate transcription, which is consistent with the larger number of repressed genes in these animals, as noted above. In contrast, the changes in cell proliferation genes were evenly distributed between positive and negative regulators of this process in Thy1-aSyn mice. In particular, alterations in the insulin-like growth factor (IGF) system, which regulates cell growth, proliferation, and apoptosis [49] may offer some clues as to the effects of SNCA on these processes. Hence, the concerted attenuation of the expression of *Igfbp6 *(Igf-binding protein 6), *Nov *(Nephroblastoma OVerexpressed, a.k.a. *Igfbp9*, or *Ccn3*), and *Ctgf *(Connective tissue growth factor, a.k.a. *Igfbp8*, or *Ccn2*) genes may increase the availability of Igf1 to activate its receptor (Igf1r), which promotes motor neurons survival [50] and thus could also contribute to neuroprotection of striatal neurons in Thy1-aSyn mice. However, the downregulation (Table 3) of glutathione peroxidase 3 (*Gpx3*), which protects cells from oxidative damage and was found decreased in mouse striatum after MPTP treatment [51], suggests that overexpression of *SNCA *may decrease cellular defenses against oxidative stress, as observed in the Thy1-aSyn mice for nigrostriatal DArgic neurons [52].

**Table 3:** α-synuclein-regulated genes associated with neurological or vascular diseases

**Hs orthologs Affymetrix **(**HGU-133A)**	Gene symbol: short description	ASO mice		Neurological disease
			
		fold change	p- value	
200862_AT	***Dhcr24***: 24-dehydrocholesterol reductase	6.1	2E-05	AD
206590_X_AT	***Drd2***: dopamine receptor D2	3.3	8E-05	Al, BD, Diab.
206713_AT	***Ntng1***: netrin G1	2.5	2E-04	Sch.
212762_S_AT	***Tf7l2***: transcription factor 7-like 2	2.1	2E-04	Diab.
205013_S_AT	***Adora2a***: adenosine A2a receptor	2.0	2E-05	Anx, ADHD,
201847_AT	***Lip1***: lipase 1, cholesterol esterase	1.9	2E-03	AD
218489_S_AT	***Alad***: aminolevulinate, δ-dehydratase	1.5	2E-04	ALS, Al, Pb Tx
203146_S_AT	***Gabbr1***: γ-aminobutyric acid receptor 1	1.5	4E-04	ADHD, Ep, Sc.
211026_S_AT	***Mgll***: monoglyceride lipase	1.5	2E-05	Al.
209702_AT	***Fto***: fat mass and obesity associated	1.5	1E-03	Diab.
212560_AT	***Sorl1***: sortilin-related receptor, LDLR class A	-1.5	3E-05	AD
210381_S_AT	***Cckbr***: cholecystokinin B receptor	-1.5	3E-03	PD, Sc, Diab.
205525_AT	***Cald1***: caldesmon 1	-1.5	1E-03	Diab.
212382_AT	***Tcf4***: transcription factor 4	-1.5	3E-03	Sc.
209189_AT	***Fos***: FBJ osteosarcoma oncogene	-1.6	6E-04	AD
203293_S_AT	***Lman1***: lectin, mannose-binding, 1	-1.6	2E-03	At, CAD, Inf. D
209101_AT	***Ctgf***: connective tissue growth factor	-1.6	7E-05	Diab.
216248_S_AT	***Nr4a2***: nuclear receptor subfamily. 4, group A, member 2	-1.7	5E-05	PD, ADHD, BD,
201348_AT	***Gpx3***: glutathione peroxidase 3 (plasma)	-1.9	2E-04	Diab.
204729_S_AT	***Stx1a***: syntaxin 1A (brain)	-1.9	2E-05	Diab.
202291_S_AT	***Mglap***: matrix Gla protein	-2.0	2E-05	At, Pb Tx
206577_AT	***Vip***: vasoactive intestinal peptide	-2.0	2E-05	MS
207848_AT	***Avp***: arginine vasopressin	-2.0	2E-05	Diab., Dement.
205352_AT	***Serpina3n***/serpin p. inhibitor, cl. A member 3n	-2.8	3E-05	Dement.
206622_AT	***Thr***: thyrotropin-releasing hormone	-8.0	1E-03	Hypertension
215775_AT	***Thbs1***: thrombospondin 1	-13.0	7E-04	At, CAD
212412_AT	***Pdzl***: PDZ and LIM domain 5	-16.0	3E-04	BD, Sc.

**Abbreviations: **D: Disease/Disorder; ADHD: Attention deficit hyperactivity D; AD: Alzheimer's D; Al: Alcoholism; ALS: Amyotrophic lateral sclerosis; Anx: Anxiety D; At: Atherosclerosis; BD: Bipolar D.; CAD: Coronary artery D; Dement: Dementia; Diab: Diabetes; Ep: Epilepsy; Hs: Homo sapiens; Inf. D: Inflammatory D; MS: Multiple sclerosis; Pb Tx: Lead toxicity; PD: Parkinson's D.; Sc: Schizophrenia

The involvement of SNCA in the pathophysiology of PD has been attributed in part to its oligomerization into protofibrils that may aggregate into insoluble inclusions, which then form Lewy Bodies [37]. Indeed, various size proteinase K-resistant SNCA inclusions have been observed in the Thy1-aSyn mice used in this study [9,10], but only small aggregates are detected in the striatum (Zhu and Chesselet, unpublished observation). It is interesting to consider that the repression of the *Tgm2c *(Tranglutaminase 2 C polypeptide) gene (Figure 1C), which mediates protein transglutamination, may prevent the formation of large aggregates in this brain region [53].

The expression of apoptosis regulatory genes was conspicuously affected in Thy1-aSyn mice. Given that neuronal cell death is not observed in the striatum of PD patients, this may provide some clues to the selective pattern of neuronal vulnerability in the face of general SNCA overexpression. Thus, the number of apoptosis genes changed in Thy1-aSyn mice reached at least 25 apoptosis genes after including 8 additional genes identified through data mining searches for apoptosis regulators (Table 2). In this study, the percentage of apoptosis genes was 11% of the total affected genes. Such prominent changes represent an apoptotic signature of the response to SNCA overexpression, indicating the usefulness of transcriptome analysis to gain insights into mechanisms influencing neurodegeneration. Hence, the 25 apoptotic genes were classified according to their effect on apoptosis, as anti-apoptotic (-), pro-apoptotic (+), and ambivalent (+ -) regulators and are shown in Figure 3C with their expression patterns. From a functional standpoint, the upregulation of anti-apoptotic genes and the downregulation of pro-apoptotic genes can contribute to neuronal survival (Figure 3C), whereas the upregulation of pro-apoptotic together with the downregulation anti-apoptotic genes can lead to cell death (Figure 3C). Such analysis reveals that SNCA overexpression caused about equal number of pro-survival and pro-death changes. However, the magnitude of pro-survival changes was more pronounced. This is particularly true for two genes that were demonstrated to have neuroprotective effects in models of AD: *Ttr *(transthyretin) and *Dhcr24 *(24-dehydrocholesterol reductase/seladin). Both were markedly induced in Thy1-aSyn mice. *Dhcr24 *is an antiapoptotic factor that protects neurons against oxidative stress and reduces amyloid formation [54-56]. *Ttr *markedly increased expression in Thy1-aSyn mice microarray was verified by qRT-PCR RNA analysis (Figure 1A, B), as well as by measuring the protein levels (Figure 2A). These verification experiments were performed in striatal tissue that was carefully dissected to avoid any contamination with choroid plexuses, which contain high levels of *Ttr *(see methods). In addition to its neuroprotective role against behavioral and biochemical effects of amyloid toxicity in murine models of AD [24], upregulation of *Ttr *was found in response to nicotine, which may protect against PD as suggested by epidemiological data [57] and in the SNc of monkeys treated with MPTP before the appearance of symptoms i.e. before the occurrence of cell death [58]. Together these data and our result suggest that Dhcr24 and Ttr may represent antiapoptotic pathway activated by SNCA overexpression. Interestingly, 3 additional apoptosis genes altered in the striatum of Thy1-aSyn mice, *Nr4a2 *(Nuclear receptor subfamily 4, group A, member 2), *Tcf7l2 *(transcription factor 7-like 2) and *Slit2 *(slit homolog 2) were similarly affected in SNc of MPTP- treated monkeys in the pre-symptomatic period [58]. Coupled to the changes in the Igf system described above, these alterations may equip striatal neurons with a battery of antiapoptotic options in response to SNCA accumulation which could explain the absence of neuronal cell death in the striatum in PD.

### Affected genes in Thy1-aSyn mice may be involved in the pathophysiology of PD

The fifth group in Table 2 encompasses biological processes that have been associated with PD, such as lipid metabolism, vascular development and neurogenesis. Studies of overexpression of wt Snca in neuronal cells suggested that Snca-polyunsaturated fatty acids (PUFA) interactions regulate neuronal PUFA levels as well as the oligomerization state of Snca [59]. Indeed, the control of vesicle recycling by Snca may be partly mediated through its ability to act as a lipid chaperone to regulate the turnover or local organization of PUFAs implicated in clathrin-mediated endocytosis [37]. Recent studies have suggested a role for Snca in brain lipid metabolism through its modulation of lipid uptake and trafficking [60]. Therefore, it is important to understand the effects of the alterations of the expression of genes involved in lipid metabolism in Thy1-aSyn mice (Figure 3E) such as the upregulation of *Srebf2 *(sterol regulatory element-binding factor 2) and *Dhcr24; *and the downregulation of *Sorl1*. *Srebf*, a transcription factor that induces cholesterol synthesis, is itself regulated by intracellular cholesterol levels [61]; *Dhcr24 *is also involved in cholesterol biosynthesis and as mentioned above protects neurons against oxidative stress and neurodegeneration [54,55]. Besides being involved in the endocytosis of APP and modulation of amyloid generation as discussed above, *Sorl1 *(a.k.a. *LR11*) is the receptor of apolipoprotein E (ApoE), which has been implicated in AD neurodegeneration [62]. Thus the expression changes in these three genes may alter cholesterol homeostasis and suggest that *SNCA *overexpression also affects lipid metabolism in the brain at the transcriptional level.

The alteration of multiple blood vessel development genes in the Thy1-aSyn mice could be relevant to the pathophysiology of PD as well. Disruption of the cerebral microvasculature may impair cholesterol efflux from the CNS and deficiencies in blood vessel development could reduce cerebral blood flow with concomitant depletion of nutrients, increase Ca2+, and elevated oxidative stress [63]. The notion that excessive SNCA interferes with vascular development is interesting, as it has been implicated in the pathogenesis of neurodegenerative diseases in conjunction with cholesterol homeostasis imbalances [62]. The pattern of expression of vascular development affected genes in Thy1-aSyn mice is shown in Figure 3E and the functions of some of them (*Ctgf, Mef2c, Tgm2*) have been discussed earlier.

The expression of genes involved in neuronal differentiation is markedly regulated in Thy1-aSyn mice as indicated by alterations in at least 20 genes that regulate this process (Table 2). The expression of the brain derived growth factor (*Bdnf*) gene, which induces proliferation and recruiting of newly born cells into the striatum [64], was down-regulated in Thy1-aSyn mice (Figure 3A). A similar decreased Bdnf expression was reported in PD striatum [65]. *BDNF *mRNA is low or absent in striatal neurons [66] but could originate in astrocytes [67] and/or cells of the subventricular zone that were included in the pooled tissue used for microarray analysis [68]. The alterations in the expression of these two genes and others neurogenesis genes raises the possibility that SNCA overexpression may affect the recruitment of newly born cells into the striatum and other brain regions and could impair adult neurogenesis, a deficit documented in similar lines of mice overexpressing SNCA [69].

### Human diseases associated with genes affected in Thy1-aSyn mice

The sixth and final group in Table 2 comprises genes altered in Thy1-aSyn mice that are associated with human diseases, namely diabetes and neurological disorders. The list of genes altered in Thy1-aSyn mice was used to find their human orthologs in the affymetrix databases; this identified 100 (of 224) orthologs, which were used to search the GAD of human diseases. The identified SNCA regulated genes associated with neurodegenerative diseases are listed in Table 3. Having identified diabetes and neurological disorders as predominant disease categories by this search, the expression and functional annotations for each of these genes was scrutinized further. A striking number of genes altered by excessive SNCA seem to be associated with metabolic diseases, most conspicuously with the diabetes phenotype, which is strongly supported by recent experimental data showing that Snca inhibited insulin secretion in β islet cells of the pancreas [70] and by the effect of excess SNCA on the expression of IGF system genes in this study. In addition, most of the neurological disorders in Table 3 share lipid imbalance as a pathophysiological feature [71]. Interestingly, a diagnosis of diabetes is more frequent in young onset PD patients than in controls [72]. Another compelling connection is the findings that the transcription factor *Tcf7l2 *was strongly increased in Thy-aSyn mice and is heavily deregulated in a PD paradigm using neuroepithelioma cells chronically exposed to rotenone (manuscript in preparation), as this gene is particularly linked to risk for diabetes and appears to be essential in β-cell functioning, since its loss of function in islets and variants of TCF7L2 in humans impair glucose-stimulated insulin secretion, which suggests that its deregulation may contribute to the susceptibility for, and pathogenesis of, type 2 diabetes [73].

### Comparison to previous transcriptome analysis

Few other studies have performed comparable analysis of gene expression in mice overexpressing *SNCA*. Yacoubian et al. [18] limited their analyses to laser-captured DArgic neurons from the SN of mice overexpressing *SNCA *under a different promoter, PDGFβ. Another major difference is that their study used both males and female mice, whereas our study was limited to male mice in view of evidence from the same group and others indicating that gender influences gene expression changes induced by SNCA overexpression in mouse SNc DArgic neurons [18], as well as the patterns of gene expression in human SNc DArgic neurons from both normal and PD brains [74,75]. Nevertheless, a functional categories analysis of Yacoubian et al. data using the updated version of DAVID that we used in this study revealed that several of the functional categories that we detected as being affected at 6 months (e.g. phosphoproteins, synapse, synaptic function, regulation of transcription, cytoskeleton, vesicles, signaling, neurogenesis and protein oligomerization) were also affected in the Yacoubian data set in 9 months old mice (albeit not the same genes). This suggests that SNCA may affect similar functional pathways in different neuronal populations, although the specific gene expression alterations, as expected, may be cell type-specific or gender-dependent. To our knowledge, changes in striatal gene expression were only examined in one other study [17]; however, in this case, *SNCA *overexpression was driven by the tyrosine hydroxylase promoter and therefore, confined to DArgic neurons innervating the striatum, not the striatal neurons themselves as in our study. Therefore, the changes observed by Miller et al. (2007) reflect changes secondary to alterations in DArgic neurons, not the effects of SNCA on striatal neurons. Nevertheless, we noticed about 20% similarity in the genes affected in the striatum compared to the changes we have observed. For example, 5 out of the 23 selected genes shown in Table 2 of Miller et al. [17] were also changed in our data set (Table S1). This is not surprising as the changes reported here likely reflect both a direct effect of SNCA on striatal neurons and changes that are secondary to SNCA induced alterations in striatal input neurons, including the nigrostriatal DArgic pathway. Previous reports of transcriptome analyses of the SNc in male PD subjects revealed a strong enrichment of pathways and cellular components relevant to PD pathogenesis that encompassed most of the functional categories associated with SNCA overexpression in this study, including synaptic transmission, neurotransmitter secretion, vesicle-mediated transport, apoptosis, synapse, cytoskeleton, signaling, and transmission of nerve impulse [74,75].

## Conclusions

**Figure 4:**
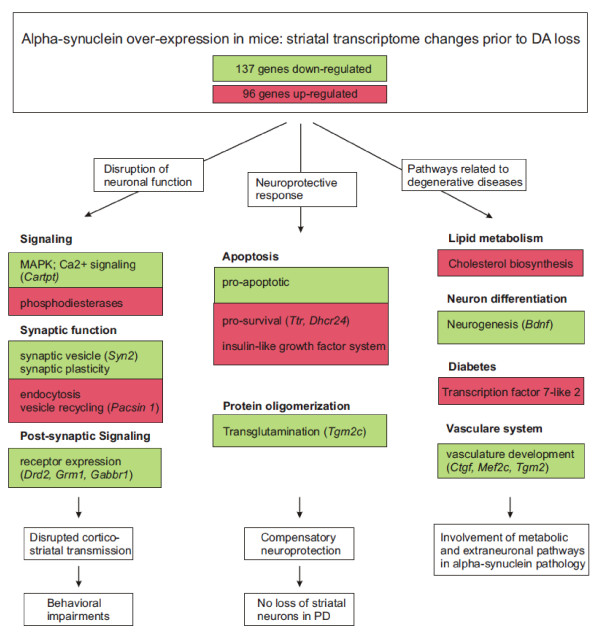
**Schematic summary of striatal gene expression changes in response to SNCA overexpression**. Green color is used to represent down-regulated and red color to represent up-regulated genes or pathways. Detailed descriptions and interpretations on gene and pathway changes can be found in the results and discussion.

A transcriptome analysis was undertaken in striatal tissue from mice overexpressing wt human SNCA under the Thy1 promoter to elucidate biological processes influenced by excessive SNCA levels. This promoter confers broad transgene expression in neurons [8]. A schematic summary of possible consequences due to striatal gene expression changes in response to SNCA overexpression is shown in Figure 4. The results from this analysis suggest that the pattern of expression of apoptotic and neuroprotective genes induced by SNCA favors cell survival, which could explain why striatal neurons do not degenerate in PD. Furthermore, alterations in the expression pattern of genes associated with synaptic function in the Thy1-aSyn mice are consistent with recent evidence indicating that excessive SNCA causes deficits in neurotransmitter release by inhibiting synaptic vesicle reclustering after endocytosis [41]; such alterations may lead to derangements at the synapses evident by the inhibition of neurotransmitter release which may impair synaptic plasticity, cause behavioral changes and contribute to neurodegeneration and eventually clinical PD.

## Methods

### Transgenic mice overexpressing human wt SNCA, and striatal tissue preparation

Animal care was conducted in accordance with the U. S. Public Health Service *Guide for the Care and Use of Laboratory Animals *and procedures were approved by the University of California, Los Angeles (UCLA), I.A.C.U. Committee. Transgenic (tg) mice overexpressing human wt *SNCA *under the Thy-1 promoter (Thy1-aSyn) created previously [8] in a mixed C57BL/6-DBA background were kept in this background by breeding mutant females with wt males. Only male mice were used in the study. The genotype of all tg and wt mice was verified by PCR analysis of tail DNA. Animals were maintained on a 12 hr light/dark cycle with free access to water and food. Six-month old male Thy1-aSyn and wt littermates were sacrificed by decapitation. For microarray analysis, whole striata from each hemisphere were immediately dissected and pooled for each brain. Tissue was permeated in RNAlater (Qiagen, Valencia, CA), frozen in liquid nitrogen, and stored at - 80°C until used for RNA preparation. For PCR verification of transcriptional changes and for protein extracts preparation, brains from 5 male Thy1-aSyn and 5 wt littermates were obtained as above but then the brains were placed in a metal brain mold with grooves to ensure reproducible cutting of thick brain slices. A first coronal cut was made with a razor blade to remove the frontal part of the brain (at bregma + 1 mm, at the anterior level of striatum). The following 1 mm coronal slice (including the striatum but no globus pallidus) was used to dissect out striatal tissue. A horizontal cut was made through the anterior commissures to exclude the nucleus accumbens. One cube of striatum was dissected out from each hemisphere, taking care not to include any corpus callosum, choroid plexus, or subventricular zone. Samples were stored at -80° until further processing.

### RNA preparation and microarray processing and data analysis

Total RNA was extracted from striata of Thy1-aSyn and wt littermates (all males) with Trizol™ (Invitrogen, Carlsbad, CA), followed by a clean-up step with RNeasy™ columns (Qiagen) and RNA integrity check using a Bioanalyzer (Agilent Tech., Foster city, CA). RNA samples were pooled, one pool representing the six control wt mice and the other representing the six *SNCA *overexpressing animals. After synthesis, using Superscript™ (Invitrogen) and labeling using the ENZO labeling kit (Affymetrix Inc., Santa Clara, CA), cRNA probes were hybridized to mouse MOE-430A GeneChip^® ^arrays (Affymetrix) following the manufacturer's protocol at the UCLA microarray core facility. Significant differential gene expression between pooled tg and wt samples was ascertained by estimation of signal log2 ratios (or fold changes in expression values), after quality control checks, data normalization and estimating expression values using the Affymetrix MAS 5.0 Software. After pairwise comparisons and filtering of this gene list using the following criteria: change p-value < 0.005 for induce genes, change p-value > 0.995 for decreased genes, signal log2 ratio > 0.6 (> 1.52 fold change), excluding probes called absent in both groups, a list of 233 differentially expressed genes was generated. We used various statistical softwares and databases to ascertain pathways affected by overexpression of *SNCA *that are associated with overrepresented genes in this gene list, including, the functional annotation tools accessible through DAVID (Database for Annotation, Visualization and Integrated Discovery, http://david.abcc.ncifcrf.gov) [21]. Additional apoptosis-related genes in the list of regulated genes were identified through data-mining searches conducted on various databases, including the University of Michigan list of apoptosis regulators (http://www-personal.umich.edu/~ino/List/AList.html). In addition, we performed literature searches on genes associated with the detected pathways, to ascertain their functions.

### Quantitative Real-Time PCR validation of results

Quantitative real-time PCR (qRT-PCR) analysis was performed with both pooled striatal RNA samples used in the microarray analysis and individual striatal RNA samples from 3 male Thy1-aSyn and 3 male control wt littermates from which cubes of striatal tissue were dissected out as described. Total RNA (500 ng) from each sample was reverse transcribed using Superscript III cDNA Synthesis Kit (Invitrogen). Primer sets for each gene (see Additional file 3, Table S2) were designed using the primer design program implemented in the Vector NTI Software (Invitrogen), and custom made by Life Technology (Invitrogen). All PCR reactions were performed using the QuantiTect SYBR GreenTM qRT-PCR kit (Qiagen), and run in triplicate in the ABI PRISM 7700 System (Applied Biosystems Inc, Fullerton, CA). All primer sets had PCR efficiencies comparable to the internal control used, *Gapdh *(glyceraldehyde-3-phosphate dehydrogenase), as determined by analysis of serial dilutions of template (10-fold); thus allowing the comparative threshold cycle Ct method to be used for relative quantification of the transcripts [76] by comparing the determined target Ct values to the Ct for *Gapdh*, thereby normalizing for small differences in starting template amounts. Data (see Additional file 4, Tables S3-ABCD) was analyzed using Prism 5.0 (Graphpad Software Inc., San Diego, CA), mean and standard errors were determined for each analyzed gene in each mice group and the one-way Mann-Whitney t-test (non-parametric) was used to estimate significance of deviations (p < 0.05) from the control samples. Pearson's test was used to quantify the magnitude and direction of the correlation between microarray and qRT-PCR assessed expression values.

### Protein extracts preparation and transthyretin measurement by ELISA

Striatal tissue samples from 6 months old Thy1-aSyn mice and wt littermates were homogenized and sonicated in a mild lysis buffer (10 mM CHAPS, 0.15 M NaCl, 0.01 M NaH2PO4, 2 mM EDTA, 50 mM NaF, 0.2 mM NaVa, 10 U/ml aprotinine, 20 U/ml DNAse I, 2 ug/ml RNAse, 1% mercaptoethanol) and centrifuged for 2 min, at 12,000 × g, at 4°C; and the soluble fraction was collected and frozen until used. The insoluble pellet was further sonicated in a stronger lysis buffer (150 mM NaCl, 10 mM NaH2PO4, 1 mM EDTA, 2% SDS, and 0.5% deoxycholate) containing the same protease inhibitor mixture. The resulting homogenate was centrifuged and the detergent-soluble fraction was collected, and frozen until used. To measure detergent-soluble fraction, a 1:100 dilution was made with PBS. To determine total amount of mouse transthyretin (Ttr), measurement of both fractions was done by ELISA as described by Purkey et al. [23]. Briefly, appropriate dilutions of antigen, pure Ttr (Sigma Co. St. Louis, MO), and striatal tissue samples (three 5-10 fold dilutions), were coated in duplicates onto 96-well Immobilon plates overnight at 4°C in PBS buffer. After washing and blocking with 0.05% Tween 20/5% non-fat powdered milk/1xPBS at 37°C for 1 h; primary goat anti-human TTR antibody (C-20: sc-8104, Santa Cruz Biotech. Inc. CA), was used at a 1:500 dilution and secondary antibody (donkey anti-goat-HRP, sc:2020, Santa Cruz) was used at a 1:1000 dilution. Detection was performed with tetramethylbenzidine (TMB) (Sigma) and stopped with sulfuric acid. Plates were read in a Kinetic microplate reader (Molecular Devices, Sunnyvale, CA) at 450 nm and analysis was performed using the SoftMax Pro LS software (Molecular Devices). Ttr concentrations were normalized to total striatal protein, as determined by Bradford assay (BioRad Labs, Hercules, CA). Data was analyzed using Prism 5.0 (Graphpad Software Inc.) mean and standard errors were determined for each group and one-way Mann-Whitney test (t test non-parametric) was used with p < 0.05 considered significant. Pearson's correlation test was used to quantify the magnitude and direction of the correlation between Ttr protein levels and *SNCA *mRNA (assessed by qRT-PCR) expression values.

## Competing interests

The authors declare that they have no competing interests.

## Authors' contributions

YCA, MFC, SF, and RHS conceived and designed the experiments; YCA and SF performed the experiments and acquired data; YCA analyzed the data and performed statistical analysis; EM, MFC and RHS contributed essential reagents and analysis tools; YCA and MFC drafted the manuscript; FR contributed to drafting the manuscript and make figures. All authors read and approved the final manuscript.

## Supplementary Material

Additional file 1 **Figure S1. Normalization scatter plot between the α-synuclein overexpressing (ASO) transgenic (tg) mice and the baseline control wild type (wt) mice illustrating the quality of the data**. Click here for file

Additional file 2 **Table S1. List of genes differentially expressed genes between α-Synuclein overexpressing (ASO) mice and wild type (wt) mice in striatal tissue at 6 months**. A gene probe was considered differentially expressed if it reported signal log2 ratio > 0.6 (> 1.52 fold change), after pairwise comparison using the Affymetrix MAS 5.0 software with change p-value < 0.005 for induce genes, and change p-value > 0.995 for decreased genes. 96 genes were upregulated (shaded in pink), whereas 137 genes showed decreased (shaded in green) expression in the ASO samples. The probes from this list were used to identify overrepresented functional categories using DAVID listed in Table 2 and illustrated in Figure 3. Click here for file

Additional file 3 **Table S2. Supplementary table listing the primer sets used for qRT-PCR analysis to corroborate microarray analysis results**. Click here for file

Additional file 4 **Table S3-ABCDE. Supplementary tables listing data used for qRT-PCR results comparisons**. (A) the qRT-PCR data used for the statistical analyses for pooled samples in Figure 1A and the microarray fold change value for each of the compared transcript (B) the t-test result for each individual transcript in Figure 1A, (C) the qRT-PCR data used for the statistical analyses for non-pooled samples in Figure 1B and the microarray fold change value for each of the compared transcript (D) the t-test result for each individual transcript in Figure 1B, and(E) The correlation analysis results between the microarray data and qRT-PCR data in Figures 1A and 1B. Additional detection statistics and fold change values for each of these probe sets and other probe sets for differentially expressed transcripts is shown in Table S1. Click here for file
