# Supplementary material for: Analysis of striatal transcriptome in mice overexpressing human wild-type alpha-synuclein supports synaptic dysfunction and suggests mechanisms of neuroprotection for striatal neurons
Source: Mol Neurodegener. 2011 Dec 13;6:83. doi: 10.1186/1750-1326-6-83 (PMC3271045; doi:10.1186/1750-1326-6-83)
Supplement: Additional file 1 — Figure S1. Normalization scatter plot between the α-synuclein overexpressing (ASO) transgenic (tg) mice and the baseline control wild type (wt) mice illustrating the quality of the data. [file 1750-1326-6-83-S1.PDF]

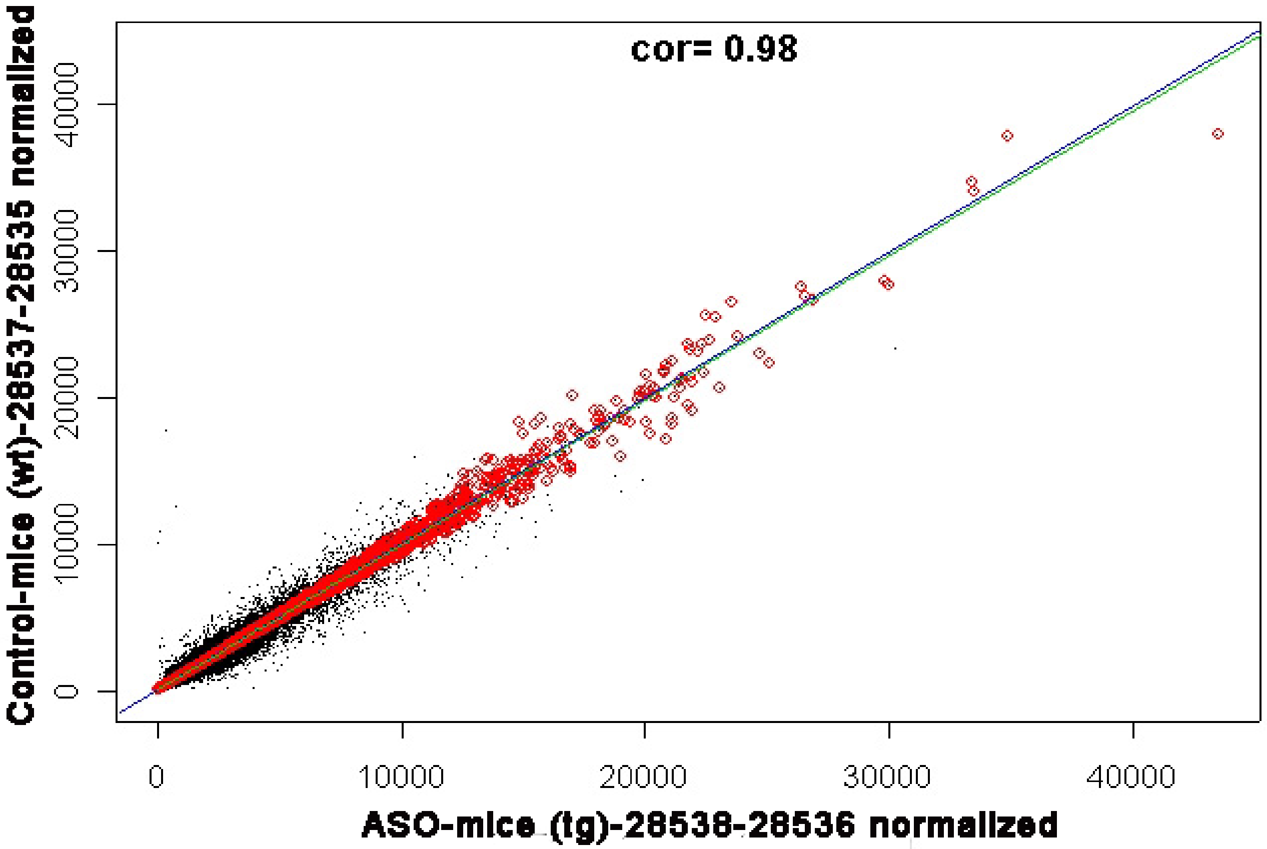

Figure S1. Normalization scatter plot between the α-synuclein overexpressing (ASO) transgenic (tg) mice and the baseline control wild type (wt) mice illustrating the quality of the data. Normalization was performed using the "invariant set" normalization method (**Li C, and Hung Wong W**., Model-based analysis of oligonucleotide arrays: model validation, design issues and standard error application. **Genome Biol 2(8):RESEARCH0032.2001**) implemented in the microarray analysis software dCHIP (http://biosun1.harvard.edu/complab/dchip/). The scatterplot is displayed in original non-log scale (M-A plots) where each point represents perfect match (PM) or mismatch (MM) probe values in the two arrays. The blue line represents the diagonal line Y = X, the red circles are the probes selected in the "invariant set", and the green curve is the running median normalization curve based on the "invariant set". Generally the deviation of the blue line and the green curve indicates the need for normalization (that is, one array is brighter than the other). Inspection of this scatterplot reveals that after normalization there was good agreement between the chips which results in data grouped along the blue line, without significant dispersion. The little scattering between wt and tg mice is indicated by the estimated high correlation coefficient between the intensities of wt and tg probe sets (cor = 0.98). Another indicator of the quality of the array data is the high number of probe sets called "Present", shown in Table 1 (manuscript) as percent present calls (% calls) values relative to the total number of probe sets on the array (All) or to the total number of differentially expressed genes (D.E.G.), as it depends on multiple factors including cell/tissue type, biological or environmental stimuli, probe array type, and overall quality of RNA. Both tg and wt samples had similar % present call values and similar mean (and median) signal intensities (Table 1), which in conjunction with this normalization data indicate the high quality of the samples analyzed.
