# Supplementary material for: Analysis of striatal transcriptome in mice overexpressing human wild-type alpha-synuclein supports synaptic dysfunction and suggests mechanisms of neuroprotection for striatal neurons
Source: Mol Neurodegener. 2011 Dec 13;6:83. doi: 10.1186/1750-1326-6-83 (PMC3271045; doi:10.1186/1750-1326-6-83)
Supplement: Additional file 2 — Table S1. List of genes differentially expressed genes between α-Synuclein overexpressing (ASO) mice and wild type (wt) mice in striatal tissue at 6 months. A gene probe was considered differentially expressed if it reported signal log2 ratio > 0.6 (> 1.52 fold change), after pairwise comparison using the Affymetrix MAS 5.0 software with change p-value < 0.005 for induce genes, and change p-value > 0.995 for decreased genes. 96 genes were upregulated (shaded in pink), whereas 137 genes showed decreased (shaded in green) expression in the ASO samples. The probes from this list were used to identify overrepresented functional categories using DAVID listed in Table 2 and illustrated in Figure 3. [file 1750-1326-6-83-S2.PDF]

Table S1. List of genes differentially expressed genes between α-Synuclein overexpressing (ASO) mice and wild type (wt) mice in striatal tissue at 6 months.

Probe Set ID	Gene Title	Gene Symbol	ENTREZ ID #	wt Signal	wt Detect	tg Signal	tg Detect	Change	Change p-value	Log Ratio	Fold change	qRT-PCR Fold change
1456182_x_at	melanoma antigen	Mela	17276	3.2	A	53.9	P	I	0.00013	2.9	7.46	
1418129_at	24-dehydrocholesterol reductase	Dhcr24	74754	21.5	A	124.4	P	I	0.00002	2.6	6.06	4.36 ± 1.07
1424454_at	transmembrane protein 87A	Tmem87a	211499	30.9	A	128.9	P	I	0.00015	2.6	6.06	
1454608_x_at	transthyretin	Ttr	22139	236.5	P	1376.9	P	I	0.00002	2.3	4.92	6.89 ± 0.95
1456471_x_at	3-phosphoglycerate dehydrogenase	Phgdh	236539	99	A	224	P	I	0.00031	1.7	3.25	3.26 ± 0.54
1428781_at	dermokine	Dmk	73712	35.3	A	88.3	P	I	0.00017	1.6	3.03	
1448600_s_at	vav 3 oncogene	Vav3	57257	8	A	36.4	P	I	0.00044	1.6	3.031	
1422847_a_at	protein kinase C, delta	Prkcd	18753	96.5	P	287	P	I	0.00002	1.5	2.83	
1419606_a_at	troponin T1, skeletal, slow	Tnnt1	21955	25.1	P	83.7	P	I	0.00027	1.4	2.64	1.89 ± 0.14
1449286_at	netrin G1	Ntng1	80883	45.7	P	129.6	P	I	0.00024	1.3	2.46	
1450828_at	synaptopodin 2	Synpo2	118449	40	A	97.7	P	I	0.00120	1.3	2.46	
1429427_s_at	transcription factor 7-like 2, T-cell specific, HMG-box	Tcf7l2	21416	10	A	43.5	P	I	0.00336	1.1	2.14	
1452913_at	Purkinje cell protein 4-like 1	Pcp4l1	66425	241.9	P	438.9	P	I	0.00019	1.1	2.14	
1420437_at	indoleamine-pyrrole 2,3 dioxygenase	Indo	15930	70	P	145.8	P	I	0.00002	1	2.00	
1421353_at	phosphodiesterase 7B	Pde7b	29863	114	P	174.3	P	I	0.00003	1	2.00	1.97 ± 0.25
1423601_s_at	Treacher Collins Franceschetti syndrome 1, homolog	Tcof1	21453	24.7	A	73.5	P	I	0.00134	1	2.00	
1424684_at	RAB5C, member RAS oncogene family	Rab5c	19345	303.6	P	572.9	P	I	0.00002	1	2.00	
1427519_at	adenosine A2a receptor	Adora2a	11540	329.3	P	631.1	P	I	0.00002	1	2.00	2.67 ± 0.27
1432490_a_at	phosphodiesterase 10A	Pde10a	23984	254.1	P	670.2	P	I	0.00002	1	2.00	
1449018_at	profilin 1	Pfn1	18643	365	P	775	P	I	0.00002	1	2.00	
1421144_at	retinitis pigmentosa GTPase regulator interacting protein 1	Rpgrip1	77945	276.6	P	484.5	P	I	0.00013	0.9	1.87	
1421276_a_at	dystonin	Dst	13518	78.1	M	144.2	P	I	0.00031	0.9	1.87	
1423140_at	lysosomal acid lipase 1	Lip1	16889	68.2	A	126.9	P	I	0.00225	0.9	1.87	
1427564_at	diaphanous homolog 2 (Drosophila)	Diap2	54004	48.2	P	94.9	P	I	0.00039	0.9	1.87	
1436713_s_at	Maternally expressed 3 (untranslated mRNA)	Meg3	17263	375.4	P	588.8	P	I	0.00225	0.9	1.87	-1.15 ± 0.08
1452624_at	leucine rich repeat transmembrane neuronal 1	Lrrtm1	74342	67.8	P	133.8	P	I	0.00024	0.9	1.87	
1416838_at	methylmalonyl-Coenzyme A mutase	Mut	17850	242.9	P	366.2	P	I	0.00019	0.8	1.74	
1417559_at	sideroflexin 1	Sfxn1	14057	74.3	P	166.7	P	I	0.00002	0.8	1.74	
1417804_at	RAS, guanyl releasing protein 2	Rasgrp2	19395	298.9	P	524.9	P	I	0.00002	0.8	1.74	
1418386_at	N-6 adenine-specific DNA methyltransferase 2 (putative)	N6amt2	68043	31.4	P	79	P	I	0.00448	0.8	1.74	
1418983_at	channel-interacting PDZ domain protein	Cipp	12695	47.4	P	91.9	P	I	0.00024	0.8	1.74	
1419429_at	ciliary neurotrophic factor receptor	Cntfr	12804	189.5	P	353.2	P	I	0.00006	0.8	1.74	
1426167_a_at	calcium/calmodulin-dependent protein kinase IV	Camk4	12326	104.2	P	159	P	I	0.00002	0.8	1.74	
1427820_at	Mus musculus cDNA clone MGC:67258, complete cds	---	---	190	P	360.4	P	I	0.00049	0.8	1.74	
1433489_s_at	fibroblast growth factor receptor 2	Fgfr2	14183	178.7	P	463.3	P	I	0.00004	0.8	1.74	
1416200_at	Interleukin 33	Il33	77125	133.9	P	193.2	P	I	0.00002	0.7	1.62	
1419635_at	RIKEN cDNA 4833420G17 gene	---	67392	35.9	P	58.4	P	MI	0.00541	0.7	1.62	
1420401_a_at	receptor (calcitonin) activity modifying protein 3	Ramp3	56089	145.7	P	331.7	P	I	0.00019	0.7	1.62	
1420707_a_at	TRAF-interacting protein	Traip	22036	37.7	A	38.9	P	MI	0.00593	0.7	1.62	
1420753_at	tolloid-like	Tll	21892	8.1	A	14.8	P	I	0.00304	0.7	1.62	
1422079_at	protein kinase C, eta	Prkch	18755	69.8	P	146.5	P	I	0.00019	0.7	1.62	
1422754_at	tropomodulin 1	Tmod1	272027	78.4	P	103.7	P	MI	0.00493	0.7	1.62	
1425700_at	glutamate receptor, metabotropic 1	Grm1	14816	59.8	P	89.1	P	I	0.00108	0.7	1.62	
1425870_a_at	Kv channel-interacting protein 2	Kcnip2	80906	232.9	P	345.1	P	I	0.00044	0.7	1.62	
1426963_at	phosphofurin acidic cluster sorting protein 2	Pacs2	217893	435	P	763.6	P	I	0.00004	0.7	1.62	
1427138_at	coiled-coil domain containing 88C	Ccdc88c	217831	62.7	P	122.1	P	I	0.00087	0.7	1.62	

1436780_at	O-linked N-acetylglucosamine (GlcNAc) transferase	Ogt	108155	329	P	432.2	P	I	0.00002	0.7	1.62	
1449261_at	pre B-cell leukemia transcription factor 2	Pbx2	18515	42.8	A	63.2	P	I	0.00203	0.7	1.62	
1449381_a_at	protein kinase C and casein kinase substrate in neurons 1	Pacsin1	23969	303.6	P	513.9	P	I	0.00003	0.7	1.62	
1450339_a_at	B-cell leukemia/lymphoma 11B	Bcl11b	58208	244.1	P	690.5	P	I	0.00002	0.7	1.62	
1451155_at	CUG triplet repeat, RNA binding protein 2	Cugbp2	329333	78.8	P	145	P	I	0.00027	0.7	1.62	
1451884_a_at	LSM2 homolog, U6 small nuclear RNA associated (S. cerevisiae)	Lsm2	27756	26.3	A	41.7	P	I	0.00120	0.7	1.62	
1452298_a_at	myosin VB	Myo5b	383411	85.7	P	170.4	P	I	0.00003	0.7	1.62	
1452353_at	G protein-coupled receptor 155	Gpr155	68526	212.6	P	327.2	P	I	0.00015	0.7	1.62	
1452544_x_at	histocompatibility 2, K region	H2-K	14972	80.9	A	104.2	P	I	0.00225	0.7	1.62	
1454043_a_at	potassium voltage-gated channel, shaker-related subfamily, β member 1	Kcnab1	16497	259.8	P	436.5	P	I	0.00002	0.7	1.62	
1415694_at	tryptophanyl-tRNA synthetase	Wars	22375	230.1	P	285.3	P	I	0.00120	0.6	1.52	
1416147_at	heat shock protein 4	Hspa4	15525	221.2	M	336.6	P	I	0.00077	0.6	1.52	
1416516_at	fascin homolog 1, actin bundling protein (Strongylocentrotus)	Fscn1	14086	106.8	A	187.6	P	I	0.00005	0.6	1.52	
1417089_a_at	creatine kinase, mitochondrial 1, ubiquitous	Ckmt1	12716	231.8	P	355.2	P	I	0.00009	0.6	1.52	1.59 ± 0.12
1417090_at	reticulocalbin	Rcn	19672	144.3	P	201.5	P	I	0.00010	0.6	1.52	
1417111_at	mannosidase 1, alpha	Man1a	17155	90.3	P	213.4	P	MI	0.00493	0.6	1.52	
1417852_x_at	chloride channel calcium activated 1	Clca1	12722	11.3	A	27.4	P	MI	0.00541	0.6	1.52	
1418573_a_at	hnRNP-associated with lethal yellow	Raly	19383	121.7	P	173	P	I	0.00183	0.6	1.52	
1418950_at	dopamine receptor 2	Drd2	13489	465.3	P	704.5	P	I	0.00008	0.6	1.52	2.07 ± 0.15
1419581_at	discs, large homolog 4 (Drosophila)	Dlgh4	13385	692.3	P	1030.2	P	I	0.00002	0.6	1.52	
1420583_a_at	RAR-related orphan receptor alpha	Rora	19883	97.1	P	146.3	P	I	0.00134	0.6	1.52	
1420885_a_at	seizure related gene 6	Sez6	20370	228.2	P	394.1	P	I	0.00007	0.6	1.52	
1421141_a_at	forkhead box P1	Foxp1	108655	204.8	P	331.6	P	I	0.00006	0.6	1.52	
1422051_a_at	gamma-aminobutyric acid (GABA-B) receptor, 1	Gabbr1	54393	319.2	P	526.4	P	I	0.00039	0.6	1.52	
1422157_a_at	integrin beta 1 binding protein 1	Itgb1bp1	16413	121.3	P	194.2	P	I	0.00069	0.6	1.52	
1423544_at	protein tyrosine phosphatase, non-receptor type 5	Ptpn5	19259	760.1	P	1169.4	P	I	0.00002	0.6	1.52	
1424467_at	pleckstrin homology-like domain, family B, member 1	Phldb1	102693	286.5	P	456.8	P	I	0.00108	0.6	1.52	
1424823_s_at	SLAIN motif family, member 1	Slain1	105439	109.4	P	154.2	P	I	0.00134	0.6	1.52	
1424985_a_at	SRY-box containing gene 10	Sox10	20665	157.4	P	310.7	P	I	0.00003	0.6	1.52	
1425075_at	GATA zinc finger domain containing 2B	Gatad2b	229542	150.8	P	262.5	P	I	0.00097	0.6	1.52	
1426336_at	calcium channel, voltage dependent, gamma-7 subunit	Cacng7	385052	111	P	177.1	P	I	0.00055	0.6	1.52	
1426458_at	sarcolemma associated protein	Slmap	83997	36.8	P	61.6	P	I	0.00004	0.6	1.52	
1426687_at	mitogen activated protein kinase kinase kinase 3	Map3k3	26406	55.2	A	86.8	P	MI	0.00493	0.6	1.52	
1426719_at	amyloid beta (A4) precursor protein-binding, family B, member 2	Apbb2	11787	153.1	P	277.5	P	I	0.00004	0.6	1.52	
1426744_at	sterol regulatory element binding factor 2	Srebf2	20788	361.4	P	538.5	P	I	0.00007	0.6	1.52	1.48 ± 0.18
1427343_at	RASD family, member 2	Rasd2	75141	988	P	1472.7	P	I	0.00005	0.6	1.52	2.03 ± 0.08
1427385_s_at	striamin	Strm	20904	126.2	P	199.5	P	I	0.00002	0.6	1.52	
1430394_a_at	ATP-binding cassette, sub-family B (MDR/TAP), member 9	Abcb9	56325	70.3	P	108.6	P	I	0.00009	0.6	1.52	
1430829_s_at	fat mass and obesity	Fto	26383	57.1	P	51.4	P	I	0.00097	0.6	1.52	
1432269_a_at	SH3-domain kinase binding protein 1	Sh3kbp1	58194	65.7	P	128.4	P	I	0.00203	0.6	1.52	
1435525_at	potassium channel tetramerisation domain containing 17	Kctd17	72844	584.4	P	1043.8	P	I	0.00031	0.6	1.52	
1436687_x_at	zinc finger protein 706; predicted gene 4002; similar to PNAS-106;	Gm10193	68036	22.9	A	31.5	P	I	0.00275	0.6	1.52	
1438511_a_at	RIKEN cDNA 1190002H23 gene	1190002H2	66214	47.2	P	57.3	P	I	0.00097	0.6	1.52	
1451188_at	WD repeat domain 26	Wdr26	226757	160.3	P	200.1	P	I	0.00002	0.6	1.52	
1451251_at	amyloid beta precursor protein (cytoplasmic tail) binding protein 2	Appbp2	66884	182	P	261.5	P	I	0.00002	0.6	1.52	
1451283_at	family with sequence similarity 114, member A2	Fam114a2	67726	82.2	P	120.5	P	I	0.00225	0.6	1.52	
1453836_a_at	monoglyceride lipase	Mgll	23945	446.6	P	707.2	P	I	0.00002	0.6	1.52	
1455191_x_at	phosphatidylinositol-4-phosphate 5-kinase, type 1 beta	Pip5k1b	18720	68.4	P	137.9	P	I	0.00249	0.6	1.52	
1455298_at	inhibitor of DNA binding 4	Idb4	15904	192.3	P	321.1	P	I	0.00275	0.6	1.52	

1460327_at	G-protein coupled receptor 88	Gpr88	64378	485.3	P	706.5	P	I	0.00004	0.6	1.52	
1415908_at	testis-specific protein, Y-encoded-like	Tspyl	22110	131.1	P	90.7	P	D	0.99851	-0.6	-1.52	
1416290_a_at	proteasome (prosome, macropain) 26S subunit, ATPase, 4	Psmc4	23996	43.5	P	55.2	P	D	0.99914	-0.6	-1.52	1.01 ± 0.06
1416724_x_at	transcription factor 4	Tcf4	21413	566.3	P	431.7	P	D	0.99998	-0.6	-1.52	
1417211_a_at	RIKEN cDNA 1110032A03 gene	1110032A(	68721	213.7	P	100.9	P	D	0.99998	-0.6	-1.52	
1417933_at	insulin-like growth factor binding protein 6	Igfbp6	16691	242.9	P	200.4	P	D	0.99945	-0.6	-1.52	
1417988_at	regulated endocrine-specific protein 18	Resp18	19711	613.3	P	443.4	P	D	0.99923	-0.6	-1.52	
1418157_at	nuclear receptor subfamily 2, group F, member 1	Nr2f1	13865	112.7	P	70	P	D	0.99979	-0.6	-1.52	
1418304_at	protocadherin 21	Pcdh21	170677	59.3	P	39.5	A	D	0.99961	-0.6	-1.52	
1419069_at	RAB guanine nucleotide exchange factor (GEF) 1	Rabgef1	56715	59.8	P	34.7	P	D	0.99979	-0.6	-1.52	
1419230_at	keratin complex 1, acidic, gene 12	Krt1-12	268482	63.1	P	31.9	A	D	0.99835	-0.6	-1.52	
1419741_at	suppressor of Ty 16 homolog (S. cerevisiae)	Supt16h	114741	565.1	P	397	P	D	0.99665	-0.6	-1.52	
1420388_at	protease, serine, 12 neurotrypsin (motopsin)	Prss12	19142	226.1	P	152.1	P	D	0.99976	-0.6	-1.52	
1422659_at	calcium/calmodulin-dependent protein kinase II, delta	Camk2d	108058	34	P	27.6	P	MD	0.99407	-0.6	-1.52	
1422825_at	cocaine and amphetamine regulated transcript (Cart) propeptide	Cartpt	27220	247.2	P	230	P	D	0.99923	-0.6	-1.52	
1423278_at	protein tyrosine phosphatase, receptor type, K	Ptprk	19272	118.4	P	87.9	P	D	0.99979	-0.6	-1.52	-3.79± 2.65
1423287_at	cerebellin 1 precursor protein	Cbln1	12404	61.2	P	56.3	P	D	0.99923	-0.6	-1.52	
1423396_at	angiotensinogen	Agt	11606	269	P	159.3	P	D	0.99665	-0.6	-1.52	
1424367_a_at	homer homolog 2 (Drosophila)	Homer2	26557	368.8	P	223.9	P	D	0.99880	-0.6	-1.52	
1424412_at	opioid growth factor receptor-like 1	Opgfrl	70155	230	P	168.4	P	D	0.99998	-0.6	-1.52	
1424737_at	thyroid hormone responsive SPOT14 homolog (Rattus)	Thrsp	21835	98.3	P	56.5	P	D	0.99835	-0.6	-1.52	
1424770_at	caldesmon 1	Cald1	109624	59.8	P	33	A	D	0.99892	-0.6	-1.52	
1424852_at	myocyte enhancer factor 2C	Mef2c	17260	1030	P	724.9	P	D	0.99914	-0.6	-1.52	1.25 ± 0.09
1424877_a_at	aminolevulinate, delta-, dehydratase	Alad	17025	142.7	P	83.6	P	D	0.99983	-0.6	-1.52	
1426258_at	sortilin-related receptor, LDLR class A repeats-containing	Sorl1	20660	714.5	P	493.1	P	D	0.99997	-0.6	-1.52	
1426427_at	tubulin tyrosine ligase-like 1	Ttll1	319953	138.1	P	91.1	P	D	0.00015	-0.6	-1.52	
1426536_at	NMDA receptor-regulated gene 2	Narg2	93697	50	P	28.1	P	D	0.99630	-0.6	-1.52	
1426636_a_at	baculoviral IAP repeat-containing 4	Birc4	11798	18.2	P	6	A	MD	0.99407	-0.6	-1.52	
1431086_s_at	protein-L-isoaspartate (D-aspartate) O-methyltransferase 1	Pcmt1	18537	145.1	P	96.7	P	D	0.99866	-0.6	-1.52	
1434020_at	PDGFA associated protein 1	Pdap1	231887	172.9	P	124.2	P	D	0.99987	-0.6	-1.52	
1436320_at	Mus musculus, clone IMAGE:4206343, mRNA	---	---	793.2	P	300.8	P	D	0.99991	-0.6	-1.52	
1436506_a_at	small nucleolar RNA host gene 6	Snhg6	73824	204.9	P	126.5	P	D	0.99725	-0.6	-1.52	
1449001_at	isovaleryl coenzyme A dehydrogenase	Ivd	56357	151.9	P	95.7	P	D	0.99696	-0.6	-1.52	
1449824_at	proteoglycan 4 (megakaryocyte stimulating factor)	Prg4	65022	68.2	P	36.6	A	D	0.99969	-0.6	-1.52	
1450193_at	hyperpolarization-activated, cyclic nucleotide-gated K+ 1	Hcn1	15165	202.3	P	153.7	P	D	0.99995	-0.6	-1.52	
1450634_at	ATPase, H+ transporting, V1 subunit A, isoform 1	Atp6v1a1	11964	1583	P	1009.8	P	D	0.99998	-0.6	-1.52	
1451308_at	elongation of very long chain fatty acids (FEN1/Elo2)-like 4	Elovl4	83603	159.9	P	106.6	P	D	0.99987	-0.6	-1.52	
1451342_at	spondin 1, (f-spondin) extracellular matrix protein	Spon1	233744	273.9	P	204	P	D	0.99725	-0.6	-1.52	
1451485_at	Luc7-like 3	Luc7l3	67684	152.5	P	73.6	P	D	0.99976	-0.6	-1.52	
1451562_at	AIG2-like domain 1	A2ld1	223267	57.7	P	44.5	A	D	0.99892	-0.6	-1.52	
1451920_a_at	replication factor C (activator 1) 1	Rfc1	19687	39.1	P	25.1	A	D	0.99593	-0.6	-1.52	
1452089_at	calcium channel, voltage-dependent, beta 4 subunit	Cacnb4	12298	227.8	P	139.3	P	D	0.99987	-0.6	-1.52	
1452189_at	WD repeat domain containing 82	Wdr82	77305	301.7	P	152.2	P	D	0.99931	-0.6	-1.52	
1452660_s_at	DNA segment, Chr 5, ERATO Doi 363, expressed	D5Ertd363	52323	243.5	P	164	P	D	0.99630	-0.6	-1.52	
1454770_at	cholecystokinin B receptor	Cckbr	12426	273.4	M	194.3	A	D	0.99696	-0.6	-1.52	-1.09± 0.29
1460230_at	synapsin II	Syn2	20965	439.8	P	287	P	D	0.99998	-0.6	-1.52	
1415922_s_at	MARCKS-like protein	Mlp	17357	367.7	P	223.6	P	D	0.99979	-0.7	-1.62	
1416250_at	B-cell translocation gene 2, anti-proliferative	Btg2	12227	192.1	P	145.1	P	D	0.99866	-0.7	-1.62	
1416405_at	biglycan	Bgn	12111	148.1	P	113	A	D	0.99989	-0.7	-1.62	

1416702_at	serine (or cysteine) proteinase inhibitor, clade I, member 1	Serpini1	20713	410.6	P	252.6	P	D	0.99997	-0.7	-1.62	
1416770_at	serine/threonine kinase 25 (yeast)	Stk25	59041	520	P	324.5	P	D	0.99992	-0.7	-1.62	
1416953_at	connective tissue growth factor	Ctgf	14219	155.3	P	115.3	P	D	0.99993	-0.7	-1.62	
1417022_at	solute carrier family 7 (cationic amino acid transporter), member 3	Slc7a3	11989	98.3	P	68.5	P	MD	0.99407	-0.7	-1.62	
1417045_at	BH3 interacting domain death agonist	Bid	12122	38.1	P	25.2	A	D	0.99725	-0.7	-1.62	
1417460_at	interferon induced transmembrane protein 3-like	Ifitm3l	80876	85.1	P	62.1	P	MD	0.99407	-0.7	-1.62	
1417461_at	CAP, adenylate cyclase-associated protein 1 (yeast)	Cap1	12331	517.7	P	270.6	P	D	0.99989	-0.7	-1.62	
1417625_s_at	chemokine orphan receptor 1	Cmkor1	12778	43.7	P	25.4	P	D	0.99866	-0.7	-1.62	
1417960_at	cytoplasmic polyadenylation element binding protein 1	Cpeb1	12877	203.5	P	140.9	P	D	0.99998	-0.7	-1.62	
1418141_at	doublecortin	Dcx	13193	349.4	P	244.7	P	D	0.99987	-0.7	-1.62	
1419034_at	casein kinase II, alpha 1 polypeptide	Csnk2a1	12995	72.4	P	42	A	D	0.99997	-0.7	-1.62	
1419228_at	elaC homolog 1 (E. coli)	Elac1	114615	76.9	P	67	P	D	0.99923	-0.7	-1.62	
1420816_at	3-monooxgenase/trp 5-monooxgenase activation protein, γ polypeptide	Ywhag	22628	250.3	P	154.5	P	D	0.99992	-0.7	-1.62	-1.73 ± 0.58
1421545_a_at	synaptic nuclear envelope 1	Syne1	64009	246.7	P	150.5	P	D	0.99981	-0.7	-1.62	
1423100_at	FBJ osteosarcoma oncogene	Fos	14281	402.5	P	345.8	P	D	0.99945	-0.7	-1.62	
1423149_at	S-phase kinase-associated protein 1A	Skp1a	21402	245.6	P	188.6	P	D	0.99998	-0.7	-1.62	
1424547_at	carbonic anhydrase 10	Car10	72605	232.2	P	114.8	P	D	0.99995	-0.7	-1.62	
1426562_a_at	olfactomedin 1	Olfm1	56177	1069	P	828.9	P	D	0.99992	-0.7	-1.62	
1427017_at	special AT-rich sequence binding protein 2	Satb2	212712	126.7	P	81.1	P	D	0.99981	-0.7	-1.62	
1427043_s_at	cytosolic ovarian carcinoma antigen 1	Cova1	209224	47.5	P	25.2	A	MD	0.99725	-0.7	-1.62	
1428130_at	lectin, mannose-binding, 1	Lman1	70361	142.4	P	73.8	P	D	0.99835	-0.7	-1.62	
1433940_at	sperm associated antigen 7	Spag7	216873	89.9	P	43.7	P	D	0.99866	-0.7	-1.62	
1436364_x_at	nuclear factor I/X	Nfix	18032	228.9	P	141.7	P	D	0.99973	-0.7	-1.62	
1450051_at	alpha thalassemia/mental retardation syndrome X-linked	Atrx	22589	110.2	P	68	P	D	0.99817	-0.7	-1.62	
1450055_at	visinin-like 1	Vsnl1	26950	1215	P	800.7	P	D	0.99990	-0.7	-1.62	
1450147_at	neuronal pentraxin receptor	Nptxr	73340	396.7	P	278.3	P	D	0.99994	-0.7	-1.62	
1450249_s_at	kinesin family member 5A	Kif5a	16572	748.9	P	477	P	D	0.99998	-0.7	-1.62	
1450852_s_at	coagulation factor II (thrombin) receptor	F2r	14062	33	P	10	M	D	0.99965	-0.7	-1.62	
1451499_at	Ca2+-dependent activator protein for secretion 2	Cadps2	320405	442.1	P	237.7	P	D	0.99989	-0.7	-1.62	
1455531_at	major facilitator superfamily domain containing 4	Mfsd4	213006	125.8	P	78.1	P	D	0.99951	-0.7	-1.62	
1460417_at	cDNA sequence AB041803	AB041803	232685	80.7	P	67	P	D	0.99665	-0.7	-1.62	
1416658_at	frizzled-related protein	Frzb	20378	86	P	43.6	A	D	0.99951	-0.8	-1.74	
1416846_a_at	PDZ domain containing RING finger 3	1110020C(	55983	71.2	P	34.7	P	D	0.99552	-0.8	-1.74	
1417705_at	OTU domain, ubiquitin aldehyde binding 1	Otub1	107260	206.2	P	119.4	P	D	0.99593	-0.8	-1.74	
1419662_at	osteoglycin	Ogn	18295	45	P	18.4	P	D	0.99923	-0.8	-1.74	
1420471_at	hypocretin	Hcrt	15171	242.9	P	118.7	P	D	0.99998	-0.8	-1.74	
1420799_at	neurotensin receptor	Ntsr	18216	56.5	P	19.2	A	MD	0.99459	-0.8	-1.74	
1425176_at	C1q-like	C1ql	227580	178.6	P	87	P	D	0.99990	-0.8	-1.74	
1425213_at	family with sequence similarity 81, member A	Fam81a	76886	1142	P	699.3	P	D	0.99998	-0.8	-1.74	
1428736_at	GRAM domain containing 3	Gramd3	107022	163.9	P	81.6	P	D	0.99993	-0.8	-1.74	
1439042_at	adenylate cyclase activating polypeptide 1 receptor 1	Adcyap1r1	11517	100.8	P	54.8	P	D	0.99775	-0.8	-1.74	
1450750_a_at	nuclear receptor subfamily 4, group A, member 2	Nr4a2	18227	132.5	P	88.8	P	D	0.99995	-0.8	-1.74	-2.03 ± 0.15
1451583_a_at	membrane magnesium transporter 2	Mmgt2	216829	75.3	P	28.7	A	D	0.99866	-0.8	-1.74	
1451620_at	phosphotriesterase related	Pter	19212	210.4	P	99.3	P	D	0.99994	-0.8	-1.74	
1452787_a_at	heterogeneous nuclear ribonucleoproteins methyltransferase-like 2	Hrmt1l2	15469	143.8	P	71.8	P	D	0.99996	-0.8	-1.74	
1460167_at	aldehyde dehydrogenase family 7, member A1	Aldh7a1	110695	271.8	P	147.2	P	D	0.99997	-0.8	-1.74	
1418188_a_at	Metastasis associated lung adenocarcinoma transcript 1	Malat1	72289	529.4	P	278.5	P	D	0.99997	-0.9	-1.87	
1421163_a_at	nuclear factor I/A	Nfia	18027	59.1	P	21.3	P	D	0.99995	-0.9	-1.87	
1424641_a_at	THO complex 1	Thoc1	225160	26.2	P	21.7	A	MD	0.99508	-0.9	-1.87	

1424659_at	slit homolog 2 (Drosophila)	Slit2	20563	140.5	P	63.9	P	D	0.99998	-0.9	-1.87	
1426114_at	heterogeneous nuclear ribonuclearprotein B	Hnrnpab	15384	258.9	P	109.9	P	D	0.99969	-0.9	-1.87	
1426856_at	hydroxysteroid dehydrogenase like 2	Hsdl2	72479	47.2	P	23.4	P	D	0.99797	-0.9	-1.87	
1437390_x_at	syntaxin 1A (brain)	Stx1a	20907	1007	P	599	P	D	0.99998	-0.9	-1.87	-1.35 ± 0.07
1438118_x_at	vimentin	Vim	22352	79	P	37.8	P	D	0.99991	-0.9	-1.87	
1438225_x_at	translocating chain-associating membrane protein 1	Tram1	72265	44.8	M	20.8	A	D	0.99552	-0.9	-1.87	
1449106_at	glutathione peroxidase 3	Gpx3	14778	219.3	P	121.5	A	D	0.99983	-0.9	-1.87	-1.67 ± 0.20
1449298_a_at	phosphodiesterase 1A, calmodulin-dependent	Pde1a	18573	159.6	P	91.3	P	D	0.99981	-0.9	-1.87	
1455900_x_at	transglutaminase 2, C polypeptide	Tgm2	21817	19.3	P	12.3	A	D	0.99630	-0.9	-1.87	
1421907_at	mediator complex subunit 1	Med1	19014	176.3	P	95.5	P	D	0.99998	-1	-2.00	-1.09 ± 0.13
1424893_at	nuclear distribution gene E-like homolog 1 (A. nidulans)	Ndel1	83431	58	P	32	A	D	0.99989	-1	-2.00	
1428664_at	vasoactive intestinal polypeptide	Vip	22353	152.6	P	83.5	P	D	0.99998	-1	-2.00	
1448416_at	matrix gamma-carboxyglutamate (gla) protein	Mglap	17313	250.1	P	118	P	D	0.99998	-1	-2.00	
1450794_at	arginine vasopressin	Avp	11998	943.9	P	384.3	P	D	0.99998	-1	-2.00	
1452730_at	ribosomal protein S4, Y-linked 2	Rps4y2	66184	116.4	P	57	A	D	0.99938	-1	-2.00	
1416711_at	T-box brain gene 1	Tbr1	21375	46.6	P	21.9	P	D	0.99965	-1.1	-2.14	
1417215_at	RAB27b, member RAS oncogene family	Rab27b	80718	27	P	10.3	A	D	0.99665	-1.1	-2.14	
1422168_a_at	brain derived neurotrophic factor	Bdnf	12064	39.4	P	20.7	A	D	0.99998	-1.1	-2.14	-2.63 ± 0.32
1422789_at	aldehyde dehydrogenase family 1, subfamily A2	Aldh1a2	19378	68.8	P	33.4	A	D	0.99751	-1.1	-2.14	
1424077_at	glycerophosphodiester phosphodiesterase domain containing 1	Gdpd1	66569	358.4	P	153	P	D	0.99923	-1.1	-2.14	
1427285_s_at	surfeit gene 4	Surf4	20932	137.8	P	59	P	D	0.99696	-1.1	-2.14	
1433685_a_at	Holliday junction recognition protein	Hjurp	381280	256.8	P	112.5	P	D	0.99998	-1.1	-2.14	
1456084_x_at	fibromodulin	Fmod	14264	45.2	P	22.5	A	D	0.99998	-1.1	-2.14	
1418271_at	basic helix-loop-helix domain containing, class B5	Bhlhb5	59058	77.4	P	49.6	P	D	0.99665	-1.2	-2.29	
1448822_at	proteasome (prosome, macropain) subunit, beta type 6	Psmb6	19175	500	P	220.7	P	D	0.99998	-1.2	-2.29	-1.65 ± 0.17
1449470_at	distal-less homeobox 1	Dlx1	13390	65.9	P	21.3	A	D	0.99961	-1.3	-2.46	
1459917_at	gametogenetin binding protein 2	Ggnbp2	217039	84.2	P	38.9	A	D	0.99998	-1.3	-2.46	
1424525_at	gastrin releasing peptide	Grp	225642	58.7	P	19.2	A	D	0.99552	-1.4	-2.64	
1426852_x_at	nephroblastoma overexpressed gene	Nov	18133	318.4	P	113.1	P	D	0.99998	-1.4	-2.64	-2.99 ± 0.40
1419100_at	serine (or cysteine) proteinase inhibitor, clade A, member 3N	Serpina3n	20716	149.8	P	34.8	A	D	0.99997	-1.5	-2.83	
1419874_x_at	zinc finger and BTB domain containing 16	Zbtb16	235320	49.2	P	17.4	A	D	0.99969	-1.7	-3.25	
1451457_at	sterol-C5-desaturase (ERG3, delta-5-desaturase, homolog S. cerevisae)	Sc5d	235293	162	P	33.2	P	D	0.99998	-1.8	-3.48	
1438390_s_at	pituitary tumor-transforming 1	Pttg1	30939	1798	P	411.3	P	D	0.99998	-1.9	-3.73	-3.53 ± 0.27
1425521_at	polyadenylate binding protein-interacting protein 1	Paip1	218693	35	P	5.8	A	D	0.99630	-2.5	-5.66	
1425090_s_at	potassium voltage gated channel, Shaw-related subfamily, member 4	Kcnc4	99738	103.3	P	16.2	A	D	0.99961	-2.6	-6.06	
1418756_at	thyrotropin releasing hormone	Trh	22044	65.1	P	8	A	D	0.99851	-3	-8.00	-2.95 ± 1.18
1425349_a_at	myelin basic protein expression factor 2, repressor	Myef2	17876	27.1	P	3.2	A	D	0.99965	-3.4	-10.56	
1421811_at	thrombospondin 1	Thbs1	21825	17.3	P	1.3	A	D	0.99931	-3.7	-12.99	
1422861_s_at	PDZ and LIM domain 5	PDZL	56376	18.6	P	0.4	A	D	0.99973	-4	-16.00	
1427242_at	DEAD (Asp-Glu-Ala-Asp) box polypeptide 4	Ddx4	13206	10.1	M	0.4	A	MD	0.99407	-4.6	-24.25	

Abbreviations: *A: Absent; P: present; M: marginal; I: Induced; D: Decreased; MD or MI: Marginally decreased of induced.* ***Description*** *: A gene probe was considered differentially expressed if it reported signal log2 ratio > 0.6 (> 1.52 fold change), after pairwise comparison using the Affymetrix MAS 5.0 software with change p-value <0.005 for induce genes, and change p-value > 0.995 for decreased genes. 96 genes were upregulated (shaded in pink), whereas 137 genes showed decreased (shaded in green) expression in the ASO samples. The probes from this list were used to identify overrepresented functional categories using DAVID listed in Table 2 and illustrated in Figure 3.*
