# Supplementary material for: Analysis of striatal transcriptome in mice overexpressing human wild-type alpha-synuclein supports synaptic dysfunction and suggests mechanisms of neuroprotection for striatal neurons
Source: Mol Neurodegener. 2011 Dec 13;6:83. doi: 10.1186/1750-1326-6-83 (PMC3271045; doi:10.1186/1750-1326-6-83)
Supplement: Additional file 3 — Table S2. Supplementary table listing the primer sets used for qRT-PCR analysis to corroborate microarray analysis results. [file 1750-1326-6-83-S3.PDF]

TableS1: List of Primer sets used for qRT-PCR analyses

Gene Symbol	Gene Name	Sense primer 5------> 3'	Antisense primer 5------> 3'	Accession #	Product length
Ttr	transthyretin	CGTTCCATGAATTCGCGGATGT	TGGTGCTGTAGGAGTATGGGCTGAG	NM_013697	99
Phgdh	3-phosphoglycerate dehydrogenase	GGAGGCTTTCCAGTTCTGCTTCTG	GTCACAGTTGAGCGGGTTTCTTCA	NM_016966	86
Pde7b	phosphodiesterase 7B	AGCAGCTGGGCTCCCTCATCTT	CAGCACACTTCAAGGCGATCTGA	NM-013875	150
Tnnt1	troponin T1, skeletal, slow	TCAATGTGCTCTACAACCGCATCAG	CAGGTCATGTCCTGGCAGTCTCACT	NM_011618	106
Adora2a	adenosine A2a receptor	GGTCCTCACGCAGAGTTCCATCTT	CAATGATGCCCTTCGCCTCATA	NM_009630	122
Meg3	Maternally expressed 3 (GTL2, imprinted maternally expressed untranslated mRNA)	CAATGGCACCCGTGTCCAACTT	GAGCCCGGACTCACTCATGAGATT	NT_166318	106
Drd2	dopamine receptor 2	AGCCACTCAGATGCTTGCCATTGT	GGTGGGATGTTGCAGTCACAGTGTA	NM_010077	105
Rasd2	RASD family, member 2	CGGCTCTCCATCCTCACAGGAGATG	GCCTCCATGGCAGGGACCTG	NM_029182	215
Psmb6	proteasome (prosome, macropain) subunit, beta type 6	ATGCTACGTATCGGGAAGGCATGA	CGCTCTACCCCTGACTCCTGAATG	NM_008946	139
Ptprk	protein tyrosine phosphatase, receptor type, k	AGGTTCCTATATGGTTGTGGACTC	TAGGATATTCAAAGTGCAGGGTTC	NM_008993	157
Pttg1	pituitary tumor-transforming 1	GCTCCTGATGATGCCTACCCAGAA	GTCATGAGAGGCACGCCATTCA	NM_01131054	128
Nov	nephroblastoma overexpressed gene	CAACAGGAATCGCCAGTGTGAGAT	GGATTTCTTGGTGCGGAGACACTTT	NM_010930	124
Med1	mediator complex subunit 1	CGGCGTCTGTGACAAATAACCCTAT	GGGCCACATCCATGAGATCATCAT	NM_013634	105
Stx1a	syntaxin 1A (brain)	AGGGCCGTGTCAGACACCAAGA	CGATGATGATGCCCAGAATCACAC	NM_016801	103
Nr4a2	nuclear receptor subfamily 4, group A, member 2	ATTGCTGCCCTGGCTATGGTCA	CAGGTAGTTGGGTCGGTTCAAACC	NM_001139509	141
Psmc4	proteasome (prosome, macropain) 26S subunit, ATPase, 4	GAAGACTATGTGGCCCGTCCAGATA	CAATGTAAGCGGTCTCACGGACAG	NM_011874	100
Mef2c	MADS box transcription (myocyte) enhancer factor 2C	CTGGCAGCAGCAGCACCTACATA	TGGCGCGTGGTGTGTTGTG	NM_001170537	198
Cckbr	cholecystokinin B receptor	TGATAATGACAGCGAGACCCAAAGC	TTGCACGTAGCAGCCATCACTGT	NM_007627	148
Dhcr24	24-dehydrocholesterol reductase	CGCAGCATCTTCTGGGAGCTC	GCACCAGCATGTCCTGCACC	NM_053272	172
Ckmt1	creatine kinase, mitochondrial 1, ubiquitous	AAAGCGTGGAACTGGAGGAGTGG	CATCGATGACGAGCTGCACCAG	NM_009897	113
Srebf2	sterol regulatory element binding factor 2	CAAGTCTGGCGTTCTGAGGAA	ATGTTCTCCTGGCGCAGCT	NM_033218	81
Trh	thyrotropin releasing hormone	GGACCTTGGTGCTGCCTTAGATTCC	CTTGGTTGGCACGTCGGCCA	NM_009426	164
Bdnf	brain derived neurotrophic factor	ATGGGTTACACCAAGGAAGGCTG	CCTTATGAATCGCCAGCCAATTC	NM_001048139	132
Gpx3	glutathione peroxidase 3	ACTGCAGAACTCCTGGGCTCACC	TGCTGACTGTGGTCCGGTGGTAC	NM_001083929	142
Ywhag	3-monooxgenase/tryptophan 5-monooxgenase activation protein, γ polypeptide	CCACTCTGATCATGCAGCTGCTC	TGTTGCCTTCACCGCCGTC	NM_018871	84
Gapdh	Glyceraldehyde-3-phosphate dehydrogenease 1	AGCAACAGGGTGGTGGACCTCA	GGATAGGGCCTCTCTTGCTCAGTG	NM_008084	102
hSNCA	synuclein, alpha (non A4 component of amyloid precursor) (human)	TTGCAGCAGCCACTGGCTTTG	GGATCCACAGGCATATCTTCCAGAA	NM_001146055	97
